# Understanding inequities in the malaria landscape of Madagascar: a scoping review of current evidence

**DOI:** 10.1186/s12936-025-05718-7

**Published:** 2026-01-14

**Authors:** Mamy Jayne Nelly Rajaofera, Wei Liu, Alphonsine Mboty Reziky, Sylvana Tomboanona, Dai Kuang, Qianfeng Xia

**Affiliations:** 1 NHC Key Laboratory of Tropical Disease Control, School of Tropical Medicine, Hainan Medical University, Haikou, 571199 Hainan China; 2 Laboratoire d’Epidémiologie Et de Biostatistique en Santé Des Populations, 401 Mahajanga, Madagascar; 3 Ecole Doctorale Nutrition-Environnement-Santé (EDNES) de l’Université de Mahajanga, 401 Mahajanga, Madagascar; 4 Ecole Doctorale Ecosystèmes Naturels, Université de Mahajanga, 401 Mahajanga, Madagascar

**Keywords:** Malaria, Madagascar, Vulnerable populations, Rural communities, Health inequities

## Abstract

**Background:**

Malaria remains a significant public health challenge in Madagascar, affecting vulnerable populations including children under five and pregnant women. Despite global progress in reducing malaria cases and deaths, Madagascar continues to experience a high burden due to inequities in access to prevention, diagnosis, and treatment.

**Methods:**

A comprehensive literature review was conducted using major academic databases including PubMed, Web of Science, and Google Scholar. A comprehensive search of literature was conducted in PubMed, Web of Science, Google Scholar, and EBSCOhost. The search focused on studies published between 2015 and 2024, supplemented by online reports. The literature was assessed for quality and relevance to malaria prevention, diagnosis, treatment interventions, and socioeconomic factors in Madagascar.

**Results:**

Disparities in malaria prevention and treatment between urban and rural areas are evident, with remote regions experiencing a higher disease burden. Geographic diversity leads to varied transmission patterns, necessitating region-specific interventions. Rural healthcare infrastructure is insufficient for timely diagnosis and treatment. Key interventions include insecticide-treated nets (ITNs), indoor residual spraying (IRS), and seasonal malaria chemoprevention (SMC). Case management primarily uses Artemisinin-based Combination Therapies (ACTs) and rapid diagnostic tests (RDTs). Social and behavior change communication (SBCC) has improved awareness but faces cultural barriers. SMC has shown promise, though logistical challenges remain. Drug resistance and diagnostic failures, along with socioeconomic inequalities, hinder effective malaria control.

**Conclusion:**

To reduce malaria’s burden in Madagascar, strengthening healthcare systems, improving supply chains, and expanding prevention efforts in underserved areas are critical. Recommendations include targeting vulnerable groups, enhancing healthcare access, and fostering international collaboration for resource allocation and equitable intervention access. Strategies should emphasize scaling up IRS, ITNs distribution, and SBCC to effectively combat malaria.

**Supplementary Information:**

The online version contains supplementary material available at 10.1186/s12936-025-05718-7.

## Background

Malaria remains a major public health challenge in Madagascar, particularly affecting vulnerable populations such as children under five and pregnant women [1, 2]. The 2024 World Malaria Report emphasizes the need to address inequities in malaria control to meet elimination goals globally [3]. The Global Strategy for Malaria Elimination (GST) provides critical guidance for countries such as Madagascar to strengthen their malaria control programmes through equitable, evidence-based interventions that ensure no population is left behind [4, 5]. Madagascar, a low-income island nation, faces persistent socioeconomic challenges, including widespread poverty, limited infrastructure, and unequal access to healthcare services [6, 7]. The health system remains under-resourced, with rural areas in particular experiencing shortages of healthcare personnel, medical supplies, and diagnostic facilities [8]. These structural factors exacerbate health inequities and hinder effective delivery of malaria interventions.

With over 31 million people at risk and nearly 27 million in active transmission zones, Madagascar’s malaria burden remains substantial [3]. The country’s diverse geography produces heterogeneous transmission patterns: lowland areas below 800 m experience perennial transmission, highland regions above 1200 m are prone to seasonal epidemics during the November to April rainy season, and mid-altitude zones between 800 and 1200 m show transitional and unstable dynamics [6, 9]. Rural communities, particularly those distant from health facilities, experience malaria incidence rates three to four times higher than urban areas [6, 10–13]. Despite progress over the past two decades, Madagascar recorded an estimated 3.97 million malaria cases and 15,974 deaths in 2023, including a 95% increase following extreme weather events such as cyclones and flooding. Children under five and pregnant women remain the most affected, and the country accounts for approximately 1.51% of the global malaria burden [3, 14–19].

Persistent gaps in malaria prevention, diagnosis, and treatment, particularly in rural and remote areas, are driven by geographic diversity, climatic variability, socioeconomic inequalities, and weak healthcare infrastructure [6–8, 14, 20, 21]. Shortages of medical supplies, limited healthcare personnel, and logistical difficulties in distributing insecticide-treated nets (ITNs), conducting indoor residual spraying (IRS), and delivering seasonal malaria chemoprevention further constrain intervention coverage. Access to timely diagnosis and artemisinin-based combination therapy (ACT) remains limited in hard-to-reach regions. These structural and operational challenges, together with ongoing conflict and violence that disrupt essential health services, intensify inequities in malaria care and undermine the effectiveness of control efforts [3, 20, 22].

Regionally, Madagascar’s malaria burden is comparable to that of other sub-Saharan African countries such as Mozambique and Tanzania [17, 24], which continue to face high incidence and mortality rates, particularly among children and pregnant women. However, countries like Ethiopia, Rwanda, and Zimbabwe have seen significant reductions in malaria cases and deaths through strong health systems, widespread ITN distribution, IRS, and improved access to diagnosis and treatment [3, 17, 23] Cabo Verde, meanwhile, has successfully achieved zero indigenous malaria cases [3, 24, 25]. In contrast, Madagascar’s unique geographic landscape, which includes endemic lowlands and epidemic prone highlands, produces heterogeneous transmission patterns that make control efforts more complex. These differences highlight the need for region specific malaria control strategies and interventions that are tailored to Madagascar’s distinct epidemiological and operational contexts while learning from the successes of better performing countries in the region.

This review aims to address this by synthesizing the available evidence on malaria in Madagascar, with a focus on identifying inequities in prevention, diagnosis, and treatment. By analyzing both current evidence and national data, the review aims to highlight key challenges, identify critical gaps, and propose actionable opportunities to strengthen the country’s malaria control response. Ultimately, the goal is to support efforts toward achieving equitable healthcare for all by addressing structural inequalities, improving resource allocation, and ensuring that no one is left behind in the fight against malaria. The review also seeks to ensure that national malaria strategies are aligned with global elimination goals, facilitating a coordinated and impactful response. Given the broad scope of the review questions, covering various aspects of malaria control such as interventions, social determinants, and health system challenges, a scoping review approach is ideal. This methodology enables the exploration of a wide range of evidence, including epidemiological studies, programme evaluations, and policy reports, while identifying knowledge gaps. Ultimately, the review will provide actionable insights, guide policy decisions, and support the alignment of national strategies with global malaria elimination goals.

## Methods

This scoping review followed the PRISMA-ScR guidelines [26] to ensure comprehensive reporting of this scoping review (Supplementary material). The review protocol and progress were discussed and recorded during team meetings, but the protocol was not registered.

### Search strategy

A comprehensive search of multiple databases was performed to identify studies related to malaria in Madagascar. The search was conducted across PubMed, Web of Science, Google Scholar, and EBSCOhost, covering publications from January 2015 to December 2024, in English and French with no publication type filters applied to ensure comprehensive retrieval of relevant literature. The PubMed search strategy (January 2025) included terms such as: ((((((((Malaria[Title/Abstract]) OR (Malaria transmission[Title/Abstract])) OR (Malaria control[Title/Abstract])) OR (Malaria vector control[Title/Abstract])) OR (Malaria prevention[Title/Abstract])) OR (Malaria diagnosis[Title/Abstract])) OR (Malaria treatment[Title/Abstract])) OR (Malaria insecticide resistance[Title/Abstract])) AND (Madagascar[Title/Abstract]).

Additional terms such as (((((((Malaria[Title/Abstract]) AND (intervention[Title/Abstract])) OR (long lasting insecticidal[Title/Abstract])) OR (indoor residual spraying[Title/Abstract])) OR (socioeconomic factors[Title/Abstract])) OR (health care[Title/Abstract])) OR (health care challenges[Title/Abstract])) AND (Madagascar[Title/Abstract]) were also used to explore the context of socio-economic and healthcare barriers in malaria control. Complete search strategies for each database are provided in Table S1.

The search was supplemented with online grey literature, including reports from WHO, the President’s Malaria Initiative (PMI), national reports, and technical documents from Madagascar’s Ministry of Health. Duplicate records were removed using Mendeley reference management software to ensure accuracy in the citation process.

### Study selection and quality assessment

Studies published between January 2015 and December 2024 were eligible for inclusion if they focused on malaria in Madagascar and addressed topics related to population disease burden, transmission dynamics, health system responses, or public health programme evaluation. Eligible studies included peer-reviewed publications, government reports, and grey literature from credible sources such as WHO and the Madagascar Ministry of Health.

Study selection was conducted by two independent reviewers (MJNR and AMR), with disagreements resolved through discussion or consultation with a third reviewer (DK). Studies were included if they addressed malaria control strategies, vector control, insecticide resistance, and socio-economic factors influencing malaria transmission. Exclusion criteria included: studies not focused on Madagascar, those lacking robust data on malaria interventions or outcomes, studies published outside the 2015–2024 timeframe, and non-peer-reviewed sources, not available in English or French. Publications with insufficient methodological transparency or relevant data were also excluded.

Quality appraisal was conducted using the Mixed Methods Appraisal Tool (MMAT) 2018 for peer-reviewed studies [27], which accommodates different study designs including qualitative, quantitative descriptive, quantitative non-randomized, quantitative randomized controlled trials, and mixed methods studies. the overall quality and credibility of sources was evaluated for governmental reports and grey literature, but did not apply a formal quality assessment tool. All quality assessment was performed independently by two reviewers and finalized through consensus.

### Data selection, extraction and synthesis

For data extraction, two reviewers (MJNR and ST) independently extracted relevant information from the included studies using a pre-piloted extraction form. Extracted data included malaria incidence, mortality, coverage of prevention tools (LLINs, IRS, ACTs, SMC), healthcare access, and socio-economic factors, with a focus on vulnerable populations such as children under five and pregnant women. A third reviewer (DK) verified the extracted data for accuracy and completeness.

### Data charting, synthesis and visualization

Trend analyses were employed to examine the current landscape of malaria control in Madagascar, drawing on data from international reports, national surveillance documents, and peer-reviewed studies. Time-series analyses evaluated trends in malaria incidence, intervention coverage, and healthcare access. Owing to methodological heterogeneity among included sources, data synthesis was narrative. Findings were organized around disease burden, control strategies, and implementation barriers. Missing data were documented but not interpolated. A structured thematic synthesis further identified recurring limitations, challenges, and inequities across key intervention domains, including geographic accessibility, healthcare delivery, population vulnerability, diagnostic capacity, community engagement, financing, climate adaptation, and multisectoral coordination. Themes were iteratively refined through cross-source comparison to ensure analytical consistency and comprehensive identification of barriers to effective malaria control. All data processing and figure generation were performed using GraphPad Prism version 9.5.

## Results

### Selection of sources of evidence

**Fig. 1:**
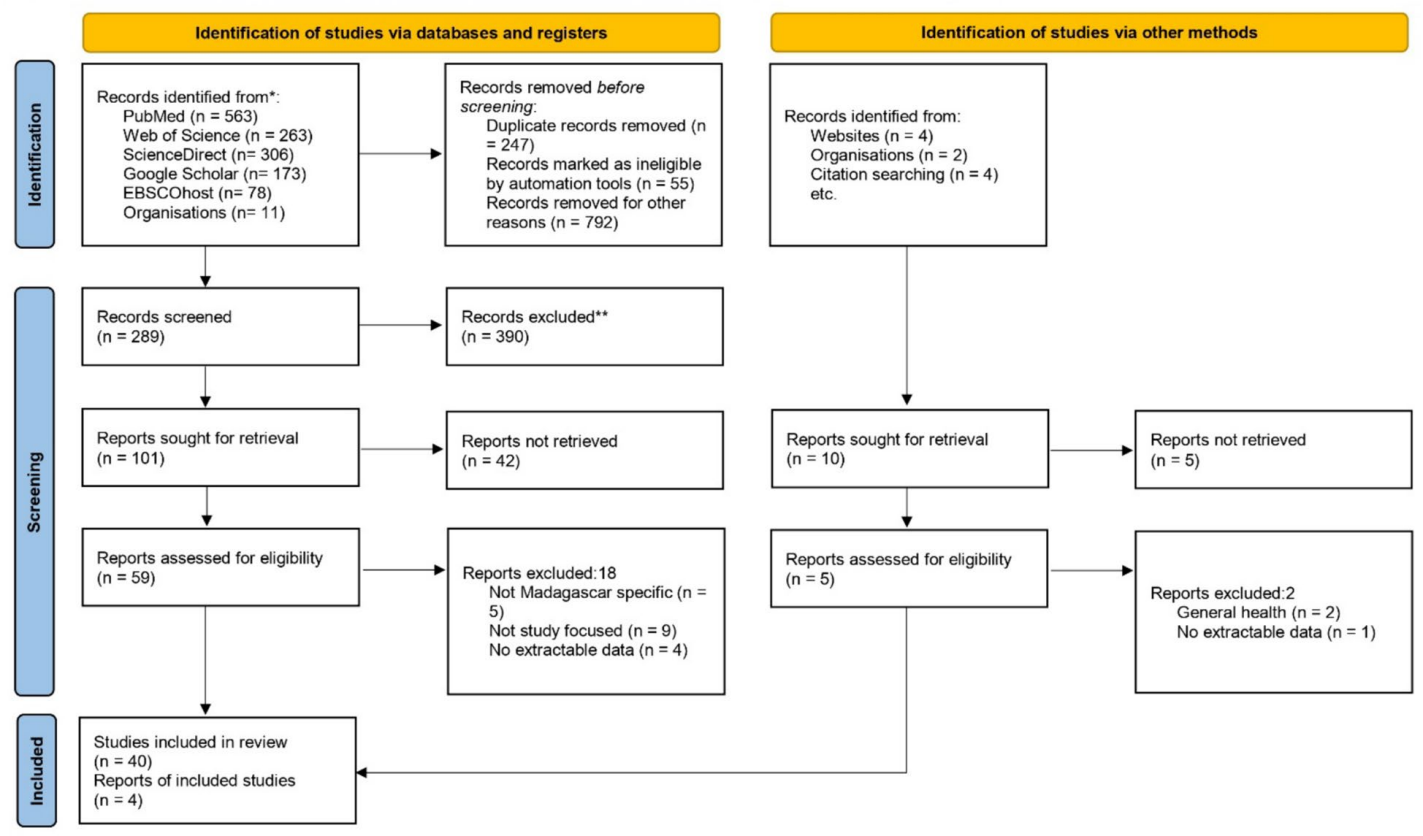
Article selection process for the scoping review following the PRISMA flow diagram methodology. The diagram illustrates the sequential stages of literature identification, screening, eligibility assessment, and final inclusion, showing the number of records at each stage and reasons for exclusion. This methodological approach ensures transparency and reproducibility in the systematic identification and selection of relevant literature for the scoping review

A total of 1,394 records were identified through database searches, including PubMed (n = 563), Web of Science (n = 263), ScienceDirect (n = 306), Google Scholar (n = 173), EBSCOhost (n = 78), and organizational reports (n = 11). Before screening, 1,094 records were removed due to duplication (n = 247), automatic exclusion (n = 55), or other reasons (n = 792). After these exclusions, 289 records were screened, and 390 were excluded. Of the 101 reports sought for retrieval, 42 could not be accessed, leaving 59 reports assessed for eligibility. 18 reports were excluded for not being Madagascar-specific (n = 5), not being study-focused (n = 9), or lacking extractable data (n = 4). An additional 10 records were identified through other sources and were sought for retrieval, of which 5 were not retrieved. The remaining 5 were assessed for eligibility, and 3 were excluded for addressing general health (n = 2) or lacking extractable data (n = 1). Ultimately, 40 studies and 4 reports (grey literature) were included in the final review. The comprehensive screening process is detailed in the PRISMA flow diagram (Fig. 1).

Of the 44 included documents, 39 peer-reviewed studies were eligible for quality assessment using the MMAT, while 5 documents were categorized as ‘‘not applicable’’ (e.g., reports or commentaries). Among the assessed studies, 29 (74.4%) were rated as high quality (meeting 4–5 MMAT criteria), 9 (23.1%) as moderate quality (meeting 2–3 criteria), and 1 (2.5%) as low quality (meeting 0–1 criteria). Overall, 97.5% of studies achieved at least a moderate quality rating, indicating a generally robust methodological standard across the included literature (Table S2).

### Characteristics of literature included

A total of 44 studies and reports were included, comprising 40 peer-reviewed articles [1, 7, 8, 11–13, 15, 16, 20, 21, 28–57] and 4 grey literature sources [3, 58–60]. The peer-reviewed studies addressed a range of malaria control topics in Madagascar, including vector control interventions (LLINs, IRS), preventive treatments (IPTp and community IPTp), case management strategies (iCCM, mCCM, Pro-CCM), diagnostic performance (RDTs, PCR, serological assays), insecticide resistance, and therapeutic efficacy. Several studies also examined socioeconomic, ecological, and occupational risk factors, in addition to spatiotemporal modelling and health system readiness. The grey literature provided additional insights into programme implementation, policy frameworks, and partner coordination, with notable contributions from PMI, WHO, and the Ministry of Health. These sources helped contextualize operational challenges and highlighted gaps in surveillance and intervention coverage. Among the peer-reviewed studies, 25 (62.5%) were observational, 8 (20%) were experimental or quasi-experimental, and 7 (17.5%) were analytical or modelling studies, reflecting a diversity of methodological approaches. A full summary of the included studies is provided in Table S3.

### Synthesis of results

This section synthesizes the key findings from the studies, highlighting the major inequities and challenges that hinder the effectiveness of malaria interventions in Madagascar. Table S4 provides a detailed overview of these factors, along with the outcomes of malaria control efforts, illustrating how they contribute to the persistence of malaria in the country.

### Epidemiology and burden of malaria in Madagascar

Malaria remains a major public health concern in Madagascar, with transmission varying by geography and season. Coastal and lowland regions experience the highest transmission, peaking during the rainy season, while mid-altitude zones have unstable transmission influenced by local climatic and environmental conditions [11, 21, 39, 42, 50]. Submicroscopic infections and multiple concurrent *Plasmodium* species confirm ongoing hidden transmission beyond routine diagnostic capacity [38, 52]. Serological surveys indicate high exposure (> 50%) in several districts [46, 50], and outbreaks correlate strongly with rainfall, flooding, and agricultural practices [13, 21, 39].

**Fig. 2:**
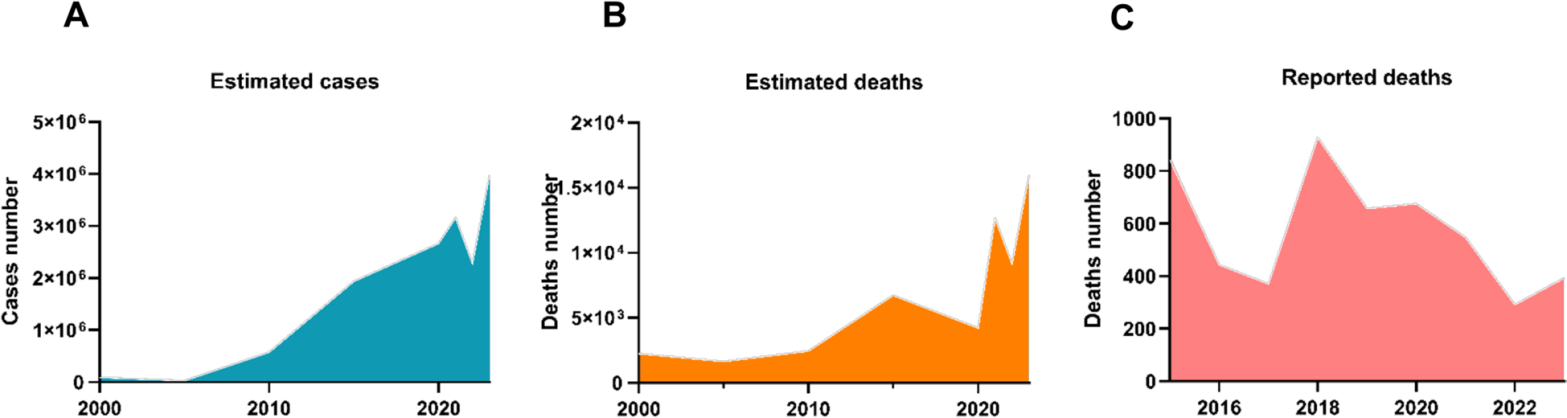
Trends of malaria in Madagascar: **A** Estimated malaria cases from 2000 to 2023, **A** Estimated deaths from 2000 to 2023, and **C** Reported malaria deaths from 2015 to 2022

Malaria cases have risen steadily since 2015, with the sharpest increases in eastern and western districts during the rainy season. Spatial analyses show parasite importation from coastal ‘‘source’’ zones sustaining transmission in highland ‘‘sink’’ areas [12, 13, 21, 39, 42]. According to the World Malaria Report 2024, 2.84 million cases were treated in Madagascar’s public sector in 2023 (Fig. 2A), a 95% rise compared to 2022. Estimated incidence increased from 88,000 (2000) to 3.97 million (2023), while mortality grew from 2,239 to 15,974 deaths (Fig. 2B) [3]. Reported deaths (n = 393) remain far lower, (Fig. 2C), reflecting underreporting and healthcare access gaps. Cyclones and flooding further intensified transmission by creating breeding sites and disrupting health services. In comparative perspective, Madagascar had a burden of 0.1274 cases per person in 2023, compared to the global average of 0.0056 and the African average of 0.2071 (Table S5). However, between 2015 and 2023, Madagascar’s malaria estimated case incidence and mortality increased by more than 105% and 137.57% respectively (Fig. 1), in contrast to the broader African region, which achieved reductions of 5% in case incidence and 16% in mortality over the same period [3], indicating that Madagascar faces distinct and escalating challenges in malaria control.

Vulnerable populations experience disproportionate disease burden. School-age children and pregnant women, who face lower access to prevention and treatment in rural areas [3, 16, 46, 48, 53, 58]. Despite the demonstrated effectiveness of IPTp and ITNs [36, 53, 56], coverage and access remain limited in remote communities, highlighting ongoing inequities in protection and maternal-child health services [3]. Additionally, vector resistance to pyrethroids and other insecticides [3, 34, 35] and sociocultural barriers to LLIN use [33, 48] further sustain transmission and reinforce disparities in malaria control.

### Progress in malaria control efforts

Madagascar has implemented several key malaria control interventions through national programmes and international partnerships, achieving notable progress in prevention and treatment coverage [31, 32, 34, 40, 53, 59]. However, these gains have not yet translated into a meaningful or sustained reduction in malaria burden, highlighting persistent gaps between intervention coverage and actual impact on transmission and mortality. The widespread adoption of RDTs has improved speed and accessibility, enabling faster detection and treatment in remote areas with limited laboratory infrastructure [1, 38, 41, 43, 44, 49, 52]. The integration PCR testing has enhanced diagnostic capabilities by detecting submicroscopic infections [37, 38], providing a more comprehensive understanding of transmission dynamics and reducing case underreporting [53]. Madagascar has implemented ACT as the first-line treatment for uncomplicated malaria, improving patient outcomes and control strategies [1, 44, 59]. The expansion of CCM has empowered community health workers to diagnose and treat malaria in underserved areas, extending service reach [7, 8].

However, significant limitation in diagnostic and treatment persist. According to the WHO, while diagnostic infrastructure exists nationwide, only 40.0% of children seeking care received a malaria diagnosis [3]. Among those diagnosed with fever and treated with an antimalarial, only 15.0% received ACT. However, when finger and heel prick testing is performed, appropriate ACT administration increases to 26.9% (Table S6), highlighting substantial underutilization of available diagnostic capacity. Critically, stockouts of ACT medicines and RDTs during outbreak periods have undermined treatment effectiveness and contributed to epidemic escalation [12]. In remote regions where resources remain most constrained, these supply chain failures disproportionately affect vulnerable populations.

Alongside treatment advances, vector control through ITNs and IRS, as well as IPTp, have been implemented as key prevention strategies. Community-based delivery of IPTp and ITNs has significantly improved preventive coverage [16, 31, 32, 53], with IPTp3 + uptake rising from 17.7% to 40.8% following community implementation [53]. Despite these gains, multiple factors have undermined prevention effectiveness. Insecticide resistance to pyrethroids and carbamates has reduced the protective efficacy of both LLINs and IRS [7, 15, 21, 32, 34] while cultural barriers and misperceptions about malaria transmission have limited consistent bed net usage [20, 33, 48] LLIN physical and chemical degradation before the expected 3-year lifespan has further reduced protection [12]. Coverage remains lower in remote areas due to logistical barriers and weak health infrastructure [21, 35, 39] IPTp coverage remains critically low, with only 11.7% of women receiving the recommended 2 + doses, primarily because health providers do not routinely offer the service [45].

Geographically, progress in malaria control remains uneven. Coastal and lowland areas continue to experience high transmission, while highland regions face seasonal epidemics, which are exacerbated by cyclones and flooding [20, 21, 39, 42]. The limited domestic funding and heavy reliance on external donors, primarily PMI/USAID and the Global Fund (Table S7), create sustainability risks and constrain long-term programme autonomy and resilience [58, 59]. This financial dependence limits the country’s ability to respond rapidly to emerging challenges and sustain consistent intervention coverage.

### Strategic approaches for malaria elimination

#### Vector control strategies

**Fig. 3:**
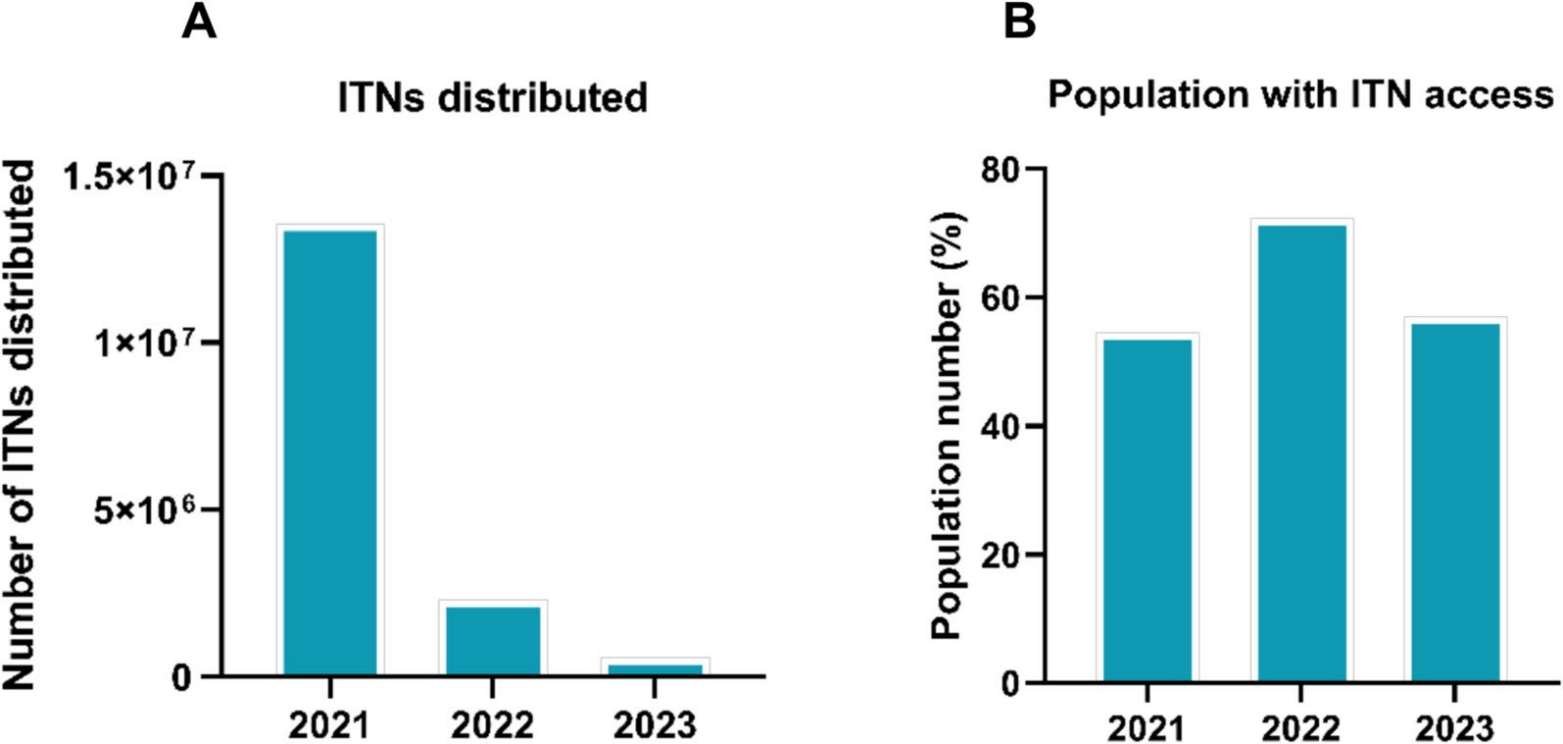
Insecticide-Treated Nets ITNs) distribution coverage in Madagascar from 2021 to 2023. **A** number of ITNs delivered, showing the number of ITNs distributed. **B** Coverage of the population with access to ITNs

LLINs remained a cornerstone of malaria control in Madagascar [31, 32, 57], with distribution strategies evolving from mass distribution campaigns (MDCs) to continuous community-based distribution (CB-CD) systems [40]. A pilot CB-CD programme in Toamasina II District demonstrated the potential of this approach, achieving 96.5% household ownership and 81.5% population access, with notably higher coverage among poorer households [36]. Continuous distribution proved superior to mass campaigns, reducing malaria cases by 14% in Toamasina sites while areas without CB-CD experienced a 12% increase [40]. However, distribution remained uneven across the country, with urban areas maintaining better access than rural regions [31, 48]. National coverage fluctuated substantially: 54.48% in 2021 (13,569,611 ITNs), 72.20% in 2022 (2,308,865 ITNs), and 56.95% in 2023 (593,425 ITNs) (Figs. 3A, B), underscoring the need for consistent distribution systems. Despite protective efficacy of 41–51% [31, 32], actual LLIN use remained suboptimal due to cultural practices prioritizing younger children and misperceptions about malaria transmission [33, 48]. In some communities, LLINs are viewed through cultural beliefs, such as being associated with marriage or death shrouds, and taboos prevent opposite-sex siblings from sharing a sleeping space. As a result, children in this age group are not allowed to use LLINs as frequently. Additionally, they are more exposed to malaria due to evening social activities and sleeping on floors without nets [50].

**Fig. 4:**
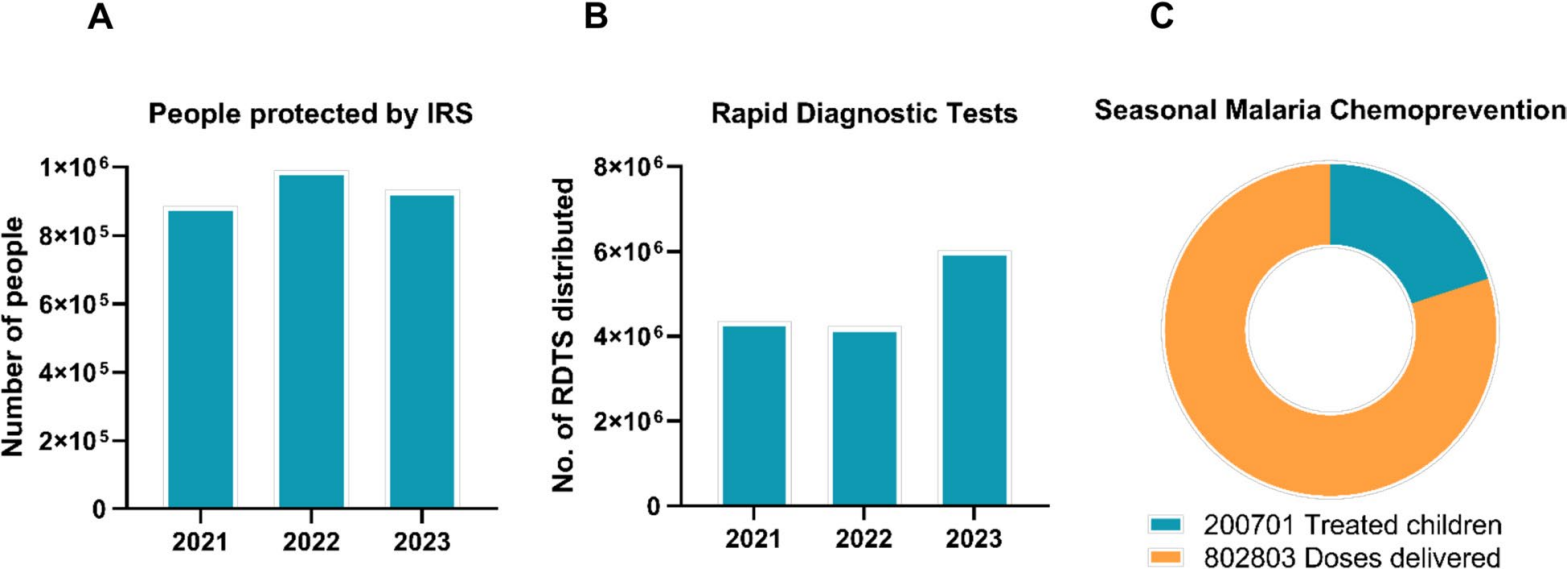
Distribution and coverage of malaria prevention and treatment commodities in Madagascar (2021–2023). **A** Number of people protected by indoor residual spraying from 2021 to 2023. **B** Distribution of Rapid Diagnostic from 2021 to 2023. **C** Number of children receiving seasonal malaria chemoprevention in 2023, represented by a pie chart showing the proportion of doses delivered (orange) and treated children (red)

IRS complemented LLIN distribution by targeting mosquito populations in high-burden areas, demonstrating impressive efficacy with 78% community protection [32, 36]. Insecticide application with Bendiocarb achieved approximately 80% mosquito mortality for up to 5 months [34], and non-pyrethroid IRS reduced malaria incidence by 30.3%, with third-year implementation proving 30.9% more effective than first-year applications [54]. Coverage of 86–90% reduced incidence by 19.7% [54]. However, Accor significant implementation barriers limited its reach. At USD 295.1 per case protected [57], IRS was considerably more expensive than LLINs. Coverage remained concentrated in high-risk regions, leaving remote and rural communities underserved. Population protected by IRS fluctuated: 885,814 people in 2021, 990,154 in 2022, and 932,715 in 2023 (Fig. 4A) [3], indicating that its reach remains limited.

#### Case management and treatment strategies

CCM has emerged as a crucial strategy for improving access to malaria diagnosis and treatment, especially in remote areas. A cluster randomized trial study showed that expanding mobile community case management (mCCM) to all age groups, rather than limiting it to children under 5, nearly tripled care-seeking for fever and malaria and increased RDT use 1.65-fold among children aged 6 to 13 years. The benefits were particularly pronounced in remote settings [8]. Baseline assessments study conducted prior to mCCM expansion revealed a malaria RDT positivity rate of 25.4% among children under 15, with higher prevalence in the 5 to 14 age group (31.8%). However, only 28.7% of children with fever sought care [49]. The integration of proactive CCM with integrated community case management (iCCM) demonstrated significant impact, reducing malaria prevalence in children under 15 (OR = 0.59) [7].

#### Diagnostic capacity and RDT distribution

RDT distribution reflected Madagascar’s strengthened focus on early detection and diagnosis. Distribution increased from 4,345,213 RDTs in 2021 to 6,021,735 in 2023 (Fig. 4B), demonstrating scaled-up efforts to ensure access to essential diagnostic tools, especially in high-risk areas. Diagnostic performance evaluations showed the SD Bioline Malaria Ag P.f/Pan RDT had 87% sensitivity and 90% specificity, with 8 false negatives but no *pfhrp2* gene deletion detected [41, 43]. Another evaluation found that sensitivity varied with parasitemia levels, with 16.3% of *P. falciparum* infections being submicroscopic [44]. Importantly, the prevalence of *pfhrp2* gene deletions remained very low at 0.6% with no dual *pfhrp2/3* deletions observed [51], indicating HRP2-based RDTs remained reliable for diagnosis.

#### Artemisinin-based combination therapy access and efficacy

ACT represents the most effective treatment for *P. falciparum* malaria, Madagascar’s predominant strain, with demonstrably improved outcomes in urban settings [3, 59]. Therapeutic efficacy has remained consistently high: artesunate-amodiaquine (ASAQ) maintained 99.7% cure rates at day 28 throughout 2012–2016, with no temporal decline [37]. Comparative trials indicate that artemether-lumefantrine (AL) clears gametocytes more rapidly than ASAQ (day 14 versus day 21) [15], suggesting potential advantages for transmission reduction. Madagascar distributed ACT medicines to treat 1,921,755 of 1,947,787 confirmed malaria cases in 2021. Treatment volumes remained stable at 1,612,781 courses in 2022 before rising substantially to 2,689,480 in 2023 (Table S8) [3]. Despite high therapeutic efficacy and improved supply volumes, equitable access remains challenging, particularly in rural areas where healthcare infrastructure and supply chains remain fragile [44].

#### Seasonal malaria chemoprevention (SMC)

In 2023, Madagascar implemented SMC for the first time, marking a significant milestone in the country’s public health efforts. SMC has proven effective in reducing malaria transmission, especially in areas with high seasonal incidence [32]. Madagascar administered 200,701 treatments in the first year and delivered 802,803 doses in total in 2023, demonstrating its commitment to combating malaria (Fig. 4C). This involvement in the SMC programme aligns with broader regional efforts to reduce malaria in SSA. In 2023, the SMC initiative treated 53,315,271 children across the region, showcasing the programme’s growing impact. Madagascar is poised to expand its participation in this initiative in the coming years, contributing to the regional goal of fighting malaria [3].

### Key challenges in malaria control

**Fig. 5:**
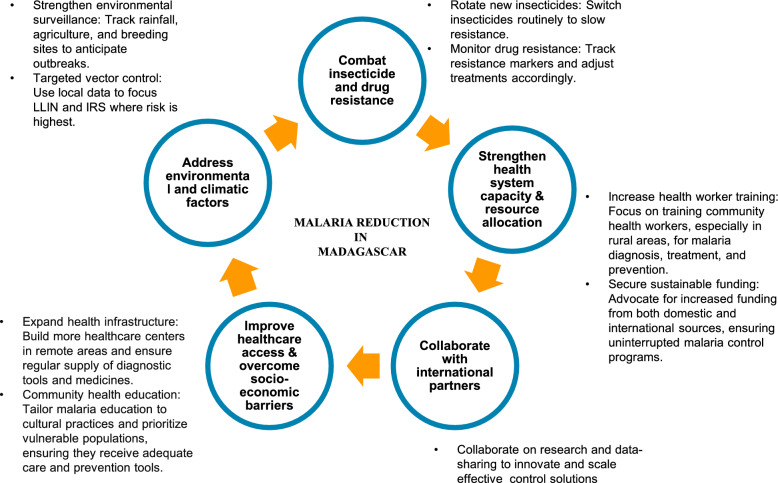
Suggests strategies for reducing malaria disparities, focusing on reaching vulnerable groups, enhancing healthcare access, broadening prevention efforts, improving supply chains, and partnering with international organizations

Malaria control in Madagascar faces several challenges, including environmental, biological, health-system, and socio-political factors (Table 1). These factors complicate efforts to reduce the disease burden and highlight the need for a comprehensive, multifaceted response (Fig. 5). The key challenges are summarized below.

**Table 1:** Summary of key challenges in malaria control in Madagascar (based on included studies)

Challenge	Description	Representative study
Environmental and climatic vulnerability	Cyclones, flooding, and rainfall create mosquito breeding sites, damage health infrastructure, and hinder access to care in rural districts. Climate variation drives seasonal outbreaks	[20–22, 32, 40]
Insecticide resistance	Resistance to pyrethroids detected in An. gambiae populations; reduced LLIN efficacy; limited alternatives threaten long-term vector control sustainability	[35, 36, 56]
Drug resistance	Low but emerging pfhrp2/3 deletions may undermine HRP2-based RDT accuracy; continued surveillance needed	[38, 53]
Healthcare access	Geographic isolation, poor transport, and inadequate infrastructure delay diagnosis and treatment; rural areas experience service gaps	[7, 8, 45, 47, 57]
Socioeconomic disparities	Poverty limits prevention and treatment access; poorer households less likely to own or use ITNs; out-of-pocket costs and supply shortages exacerbate inequities	[30, 31, 34, 37, 50, 59]
Behavoral and cultures barriers	Misperceptions about malaria cause and prevention; LLIN underuse by older children and low uptake of IPTp; local beliefs influence treatment-seeking	[34, 37, 50]
Operational and data management	Incomplete reporting, inconsistent LLIN/IRS coverage, and weak monitoring systems hinder evaluation and resource allocation	[37, 56–58]
Funding dependence	Heavy reliance on external donors (PMI, Global Fund, CRS) risks program sustainability; limited domestic investment in malaria control	[57, 58, 60]

pfhrp2/3 – Plasmodium falciparum histidine-rich protein 2 and 3 genes, HRP2: Histidine-Rich Protein 2, RDT: Rapid Diagnostic Test, LLIN: Long-Lasting Insecticidal Net, IRS: Indoor Residual Spraying, IPTp: Intermittent Preventive Treatment in Pregnancy, ITNs: Insecticide-Treated Nets PMI: President’s Malaria Initiative, CRS: Catholic Relief Services

#### Climate change and environmental challenges

Environmental and climatic factors play a significant role in malaria transmission dynamics across Madagascar. Increased rainfall has been directly linked to malaria outbreaks by creating favourable mosquito breeding conditions [21]. This environmental risk is amplified by aquatic agriculture, which serves as a strong predictor of *Anopheles* larvae presence [20]. However, the interplay between these ecological and socioeconomic factors varies considerably by region, creating distinct malaria risk patterns throughout the country [20, 39].

Malaria transmission exhibits extreme spatial heterogeneity, with prevalence varying more than tenfold between nearby communities. Coastal areas, particularly the west coast, bear the highest burden, with prevalence rates as high as 29.4%, while in southeastern regions, nearly 50% of households harbour multiple infections [50]. Between 2010 and 2014, malaria prevalence increased substantially, with the highest incidence concentrated in the East and West regions [11, 39]. Temporal patterns further complicate malaria control. The eastern coast experiences earlier seasonal peaks, though national transmission generally occurs from January to July [12, 39]. Seasonal impacts are dramatic, with adjusted malaria incidence reaching four times higher during peak transmission periods compared to off-season months [47]. In addition, population mobility adds complexity by creating parasite importation hubs in the Central highlands, while coastal areas serve as primary infection sources [42]. These seasonal transmission dynamics pose significant challenges for implementing effective, year-round control strategies [42].

#### Insecticide and drug resistance

Insecticide resistance poses a critical threat to vector control effectiveness. *Anopheles gambiae*, *Anopheles funestus*, and *Anopheles mascarensis* have demonstrated widespread resistance to pyrethroid and carbamate, significantly reducing the efficacy of LLINs and IRS in multiple regions, although susceptibility to pirimiphos-methyl remains [35]. Experimental trials underscored the need for alternative insecticides [34].

Additionally, the identification of *Anopheles coustani* as a major malaria vector represents an additional and significant challenge to conventional control strategies. This vector exhibits high abundance in certain districts (up to 45.2% of mosquito populations in areas like Ankazobe), opportunistic indoor/outdoor biting behaviour, and confirmed *Plasmodium* infection, with human biting rates reaching as high as 86.1% in some areas [28]. Critically, the zoophilic (animal-preferring) and exophilic (outdoor-resting) behaviours of *An. coustani* enable it to sustain residual transmission even in areas with high LLIN and IRS coverage. These indoor-focused interventions fail to reach outdoor-biting and animal-feeding mosquitoes, limiting the impact and cost-effectiveness of LLINs and IRS in regions where *An. coustani* predominates [47, 51].

Drug resistance remains a concern, although it is currently low. *Pfhrp2* gene deletions are minimal at 0.6% with no dual *pfhrp2/3* deletion [43, 51], but these mutations can lead to false-negative RDT results, delaying treatment [41]. Emerging mutations in *pfcrt* and *pfmdr1* genes indicate growing drug tolerance, with artemisinin resistance markers detected in 3–7% of samples from high-transmission areas [34, 35, 51].

#### Barriers to healthcare access and socio-economic disparities

Socio-economic factors profoundly shaped malaria burden and intervention access. Education and wealth strongly influenced malaria knowledge, prevention practices, and prevalence [29], with infections concentrated among rural, poor populations and children aged 6 to 14 [31]. High-risk groups, including rice workers, miners, mobile vendors, and students, faced multiple barriers: travel to endemic areas, overnight stays, lack of prevention tools, and limited access due to cost and distance to health facilities [55].

Geographic isolation and inadequate infrastructure created stark access disparities. Urban and semi-urban areas enjoyed better intervention coverage, while rural and remote regions confronted significant logistical challenges. These disparities translated into poor care-seeking behavior, with only 28.7% of febrile children receiving medical attention [49]. Community surveys revealed that most infections remained undetected, with 13.8% PCR positivity compared to only 4.1% RDT positivity [38]. School-based serosurveys further demonstrated this hidden burden: while RDT positivity was merely 0.5%, seroprevalence ranged from 17.9% to 59.7% [46], indicating a substantial reservoir of submicroscopic infections. Village location, bed net ownership, fever history, and household infection status were key risk determinants [38].

Although long-lasting insecticidal nets (LLINs) are perceived positively, their use among children aged 5–15 remains low. This is primarily due to the cultural prioritization of under-fives and pregnant women, as well as specific cultural beliefs that associate LLINs with marriage or death shrouds. Additionally, taboos preventing opposite-sex siblings from sharing a sleeping space further limit the use of nets in this age group. Consequently, children aged 5–15 are more exposed to malaria, particularly due to their evening social activities and the common practice of sleeping on floors without nets [48]. Moreover, LLINs are not predominantly seen as tools for malaria prevention but are instead used for protection from insect nuisance, cold, and privacy. Malaria is often misunderstood as a simple fever rather than a mosquito-borne disease, leading to inconsistent bed net use and irregular malaria prevention practices [33, 48]. Biomedical health messages were poorly aligned with local beliefs, creating a gap between official health education and community understanding. As a result, malaria messages were largely ineffective, with information spreading more effectively through informal channels and rumors. These cultural practices and misconceptions contribute to the underserved status of children over five, who are often left without nets when they are in short supply[33].

#### Health system weaknesses

Health system weaknesses further undermine malaria control efforts. Readiness assessments have shown that 25% of health facilities lack RDTs, only 43% of fever patients are tested, and 24% of community health volunteers no longer treat fever cases, with average district readiness scores of only 52 out of 100 [1]. These health system challenges were exacerbated by political instability following 2009, which disrupted malaria control programmes, reduced international funding, and weakened surveillance infrastructure [55]. This period of instability contributed to the deterioration of control gains achieved in the previous decade. Data quality also remains a significant concern, with data completeness ranging from 43 to 68%, timeliness from 85 to 95%, and source documentation accuracy from 48 to 59% across regions [57]. Major data gaps affecting 60–75% of reporting hindered effective control planning [3]. Geographic analysis estimated 80% underreporting of malaria cases, with adjusted incidence four times higher during high transmission seasons [47], highlighting critical surveillance weaknesses.

#### Intervention coverage and resource constraints

Maintaining consistent intervention coverage has proven difficult due to significant resource constraints. LLIN distribution fluctuated substantially, with mass distribution campaigns showing diminishing effects after 1–2 years [40]. Additionally, LLIN durability remains a challenge, as nets degrade physically and chemically before their expected 3-year lifespan, reducing their protective efficacy and necessitating more frequent replacements than originally planned [12]. Outbreak investigations have linked malaria epidemics to reduced LLIN integrity, declining insecticide activity, drug stockouts, and increased rainfall [21].

IRS coverage remains limited due to its high costs (USD 427.6–546.3 per DALY compared to USD 45.3–85.4 for ITNs) [56], concentrating protection in high-risk regions while leaving remote areas underserved [58]. The significant cost differential between interventions influences resource allocation decisions, prioritizing more cost-effective options like ITNs in resource-constrained settings. This has led to uneven geographic coverage, with ITNs being favoured over IRS despite IRS's higher cost and lower cost-effectiveness. In the low-transmission district of Ankazobe, IRS was implemented despite ITNs proving more efficient, raising questions about the optimal allocation of resources. Furthermore, IPTp coverage remains critically low, with only 11.7% of women receiving the recommended 2 + doses, primarily due to the lack of routine service delivery and community-level barriers [16]. These coverage gaps reflect broader resource constraints within the malaria control programme, which requires substantial funding, with annual costs for the NMP estimated at USD 2.0 per capita per year [56]. Balancing effectiveness with cost-efficiency while ensuring sustainable financing remained challenging. Despite international support, including from the President’s Malaria Initiative which trained over 204,600 health workers [3, 60], resource constraints limit comprehensive coverage. The WHO 2024 report confirmed that while ITNs and IRS were widely used, access gaps remained key challenges [59].

### Inequities in malaria control in Madagascar

Persistent inequities continue to drive disparities in malaria burden and control outcomes across Madagascar, spanning geographic, socioeconomic, gender, and health system dimensions. Geographically, rural and remote communities bear the heaviest malaria transmission yet have the least access to diagnostic tools, treatment, and preventive measures like LLINs and IRS [7, 8, 21, 39, 42, 44, 50]. These challenges are compounded by socioeconomic disparities: poorer households consistently show lower rates of bed net ownership and use, reduced healthcare access, and heightened exposure due to substandard housing and agricultural livelihoods [16, 20, 29, 30, 32, 48, 50]. Gender inequities remain stark, with pregnant women facing elevated malaria risks yet experiencing persistently low uptake of IPTp and limited access to antenatal care [16].

These barriers are often reinforced by social norms and constrained decision-making power [40, 53]. Health system inequities are equally pronounced: rural populations frequently live far from health facilities, encounter frequent drug stockouts, and depend on overstretched community health workers, all of which contribute to delays in diagnosis and treatment [7, 8]. Adding to these challenges, biological and ecological inequities arise from spatial variation in insecticide resistance, which undermines vector control effectiveness in certain regions [3, 34, 35]. Finally, conflict and displacement disrupt malaria service delivery and prevention efforts in affected areas, further marginalizing vulnerable populations and deepening inequitable access to care [3, 40, 55, 58]. Moreover, data and surveillance inequities hinder effective malaria control efforts. Inconsistent data quality, underreporting, and poor timeliness of malaria case reporting make it difficult to accurately track malaria incidence and direct resources effectively, particularly in high-transmission areas [57]. These intersecting inequities impede progress toward malaria elimination in Madagascar and highlight the urgent need for tailored, context-specific interventions that target these inequities, ensure equitable access to resources, and reach all population groups. Further details on each inequity and its impact on malaria control efforts are presented in Table S4.

## Discussion

This review synthesizes findings from 44 studies and reports, revealing that malaria in Madagascar remains a persistent public health challenge shaped by geographic heterogeneity, biological threats, and structural inequities. Despite the national scale-up of interventions and strong external support, the country’s malaria burden has not declined at the same rate as much of SSA, highlighting implementation and equity gaps that continue to hinder elimination progress.

Malaria transmission in Madagascar shows marked spatial and temporal variation, with high endemicity in coastal and lowland regions and unstable, epidemic-prone transmission in the highlands [20, 21, 39, 42, 50]. These localized patterns differ from the more uniformly high transmission typical of many SSA settings. The country’s sensitivity to climatic factors such as cyclones, heavy rainfall, and temperature fluctuations further amplifies these disparities. For instance, post-cyclone flooding in 2023 led to nearly twice as many malaria cases, exposing major weaknesses in surveillance and response capacity. While several SSA countries have strengthened resilience through early warning systems and climate-informed malaria models, Madagascar’s health system remains inadequately prepared for these climate-driven surges [61]. This highlights the urgent need for climate adaptation strategies integrated into malaria control programmes to address seasonal variations and extreme climate events.

Although core malaria interventions such as LLINs, IRS, and intermittent preventive treatment in pregnancy (IPTp) have expanded, coverage remains inconsistent and inequitable [1, 2]. Compared with several SSA countries that achieved steady gains through universal coverage campaigns, Madagascar continues to face logistical and geographic barriers that limit access in rural and remote communities [34, 35, 54, 62]. Studies reveal periodic declines in LLIN distribution, fluctuating IRS coverage, and persistent gaps in IPTp uptake among pregnant women [16, 31, 32, 53]. These findings point to weaknesses in supply chain management, programme continuity, and supervision rather than to limitations in intervention effectiveness. In contrast, countries such as Rwanda and Zambia have maintained higher coverage through stronger local governance and coordinated donor support, achieving measurable reductions in both malaria incidence and mortality [3, 63].

The observed decline in annual ITN distribution while population access remained relatively stable warrants clarification. This pattern reflects the temporal lag inherent in ITN programmes: population access is a cumulative measure capturing nets distributed over multiple years, whereas distribution figures reflect only new nets delivered in a given period. Given that LLINs retain efficacy for approximately 2–3 years under field conditions [21, 36, 40], households continue to benefit from nets received in prior distribution campaigns even when current-year distribution declines. However, this buffering effect is temporary and unsustainable. Without consistent replenishment to replace aging nets, access will inevitably decline as existing nets become torn, lose insecticidal efficacy, or are discarded. This underscores the need for predictable, continuous ITN distribution systems rather than sporadic mass campaigns, particularly in Madagascar where logistical challenges and funding gaps have led to irregular replacement cycles [40].

Reproductive, maternal, newborn, and child health (RMNCH) platforms, despite achieving broad coverage, remain insufficiently integrated with malaria interventions, resulting in missed opportunities for IPTp delivery, LLIN distribution, and malaria education [1, 2, 62]. Madagascar’s immunization programmes, which consistently reach over 85% coverage for key vaccines [64], are rarely used as channels for malaria prevention, despite their potential for efficient, cost-effective outreach [1, 64]. Likewise, water, sanitation, and hygiene (WASH) initiatives operating in rural areas present untapped opportunities for environmental management and community-based education. Greater coordination between WASH, RMNCH, and malaria programmes could enhance intervention reach and long-term sustainability [65, 66].

Biological resistance poses an additional threat consistent with regional trends across SSA. Insecticide resistance among *An. gambiae*, *An. funestus*, and *An. mascarensis* has been documented across Madagascar, weakening vector control effectiveness [34, 35]. Similarly, early molecular markers of artemisinin tolerance, including *pfcrt* and *pfmdr1* mutations, have emerged [33]. These findings mirror regional patterns, where rising insecticide and drug resistance risk reversing hard-won progress [51]. Continuous molecular surveillance, rotation of insecticide classes, and diversification of LLIN products are urgently needed to preserve efficacy.

Madagascar’s dependence on external financing mirrors the structural vulnerability faced by many SSA countries. Nearly all malaria programme funding between 2021 and 2023 originated from the Global Fund, PMI, and other donors, while domestic contributions remained minimal [3, 58]. Compared to SSA averages, where donor reliance remains high but national co-financing has gradually increased, Madagascar’s limited budgetary allocation to health (5.9% in 2022) threatens long-term sustainability. Economic shocks or shifts in donor priorities could jeopardize essential malaria control operations, underlining the importance of increasing domestic fiscal commitment and integrating malaria control into broader health system strengthening initiatives.

Overall, Madagascar’s situation reflects a wider regional pattern where malaria persists in contexts of inequality, environmental instability, and health system fragility. Yet Madagascar’s challenges are more geographically distinct and climate-sensitive. While countries such as Rwanda, Cabo Verde, and Eswatini are advancing toward elimination through strong governance and adaptive surveillance, Madagascar’s progress remains constrained by fragile infrastructure and uneven coverage [24, 25]. Moving forward, integrating malaria control with climate adaptation, decentralizing health management, and expanding domestic financing are critical for sustainable progress.

## Limitations

This review is limited by heterogeneity among included studies, most of which were cross-sectional or operational. Variations in study design, diagnostic methods, and geographic scope hinder direct comparison. Surveillance data likely underestimate malaria burden due to underreporting and incomplete case confirmation. Despite these constraints, triangulating peer-reviewed and grey literature provides a robust understanding of national trends and key determinants.

## Conclusion

Madagascar’s malaria situation reflects the complex interplay between ecological diversity, health inequity, and systemic weakness. Although notable progress has been made through vector control, preventive treatment, and community participation, persistent regional disparities, growing resistance, and recurring climate shocks continue to hinder elimination efforts. To accelerate malaria control, context-specific strategies are essential, focusing on strengthening surveillance, building climate-resilient health systems, and ensuring equitable access to prevention and care. Strengthening healthcare systems, improving supply chains, and expanding interventions in underserved areas are critical. Recommendations include targeting vulnerable populations, enhancing healthcare access, and fostering international collaboration to improve resource allocation and intervention equity. Scaling up IRS, ITN distribution, and SBCC will be key to effectively reducing malaria’s burden and achieving the goal of elimination.

## Supplementary Information

Supplementary material 1 Table S1. Search protocols and strategies employed across different databases in this scoping review

Supplementary material 2 Table S2. Quality Assessment Summary

Supplementary material 3 Table S3. Characteristics and key insights of included studies and reports on malaria control in Madagascar in this review

Supplementary material 4 Table S4. Key Findings from the literature on malaria control in Madagascar. This table summarizes the main findings from the studies included in this review, addressing the diverse factors influencing malaria control in Madagascar. It includes data on intervention effectiveness, geographic and seasonal transmission patterns, and socio-economic and health system challenges that impact malaria prevention and treatment efforts across the country

Supplementary material 5 Table S5. Estimated malaria cases and deaths, along with rates per population for global, African countries, and Madagascar in 2023

Supplementary material 6 Table S6. Coverage of treatment-seeking behavior for fever, diagnosis rates, and ACT usage among children under 5 years old based on the most recent household survey in Madagascar

Supplementary material 7 Table S7. Malaria control funding in Madagascar, 2021–2023

Supplementary material 8 Table S8. Distribution of commodities and coverage for malaria treatment in Madagascar, 2021-2023, with a focus on ACTs

## Data Availability

The datasets generated and/or analyzed during the current study are available from the corresponding author on reasonable request.

## Abbreviations

ACT: Artemisinin-based Combination Therapy
DHS: Demographic and Health Survey
GST: Global Strategy for Malaria Elimination
iCCM: Integrated Community Case Management
IPTp: Intermittent Preventive Treatment during pregnancy
IRS: Indoor Residual Spraying
ITNs: Insecticide-Treated Nets
LLINs: Long-Lasting Insecticidal Nets
mCCM: Mobile Community Case Management
MMAT: Mixed Methods Appraisal Tool
NMP: National Malaria Programme
Pfhrp2/3: *Plasmodium falciparum* Histidine-rich protein 2/3
PMI/USAID: President’s Malaria Initiative/United States Agency for International Development
RDTs: Rapid Diagnostic Tests
RMNCH: Reproductive, maternal, newborn, and child health
SMC: Seasonal Malaria Chemoprevention
SSA: Sub-Saharan Africa
UNICEF: United Nations Children’s Fund
WHO: World Health Organization
WASH: Water, sanitation, and hygiene
