# Supplementary material for: Understanding inequities in the malaria landscape of Madagascar: a scoping review of current evidence
Source: Malar J. 2026 Jan 14;25:91. doi: 10.1186/s12936-025-05718-7 (PMC12888438; doi:10.1186/s12936-025-05718-7)
Supplement: Supplementary file 1 — Supplementary material 1 Table S1. Search protocols and strategies employed across different databases in this scoping review [file 12936_2025_5718_MOESM1_ESM.docx]

**Table S1.** Search protocols and strategies employed across different databases in this scoping review

| **Database** | **Search protocol** |
| --- | --- |
| **PubMed** | 1. ((((((((Malaria[Title/Abstract]) OR (Malaria transmission[Title/Abstract])) OR (Malaria control[Title/Abstract])) OR (Malaria vector control[Title/Abstract])) OR (Malaria prevention[Title/Abstract])) OR (Malaria diagnosis[Title/Abstract])) OR (Malaria treatment[Title/Abstract])) OR (Malaria insecticide resistance[Title/Abstract])) AND (Madagascar[Title/Abstract]) 2. (((((((Malaria[Title/Abstract]) AND (intervention[Title/Abstract])) OR (long lasting insecticidal[Title/Abstract])) OR (indoor residual spraying[Title/Abstract])) OR (socioeconomic factors[Title/Abstract])) OR (health care[Title/Abstract])) OR (health care challenges[Title/Abstract])) AND (Madagascar[Title/Abstract]) |
| **ScienceDirect** | 1. Malaria OR ''Malaria transmission'' OR ''Malaria control'' OR ''Malaria vector control'' OR ''Malaria prevention'' OR ''Malaria diagnosis'' OR ''Malaria treatment'' OR ''Malaria insecticide resistance'' Year 2015-2024; Title, abstract, Keywords: Madagascar 2. ''Malaria intervention'' OR ''long lasting insecticidal'' OR ''indoor residual spraying'' OR ''health care'' OR ''health care challenges'' Year 2015-2024; Title, abstract, Keywords: Madagascar |
| **Google Scholar** | 1. Malaria OR ''Malaria transmission'' OR ''Malaria control'' OR ''Malaria vector control'' OR ''Malaria prevention'' OR ''Malaria diagnosis'' OR ''Malaria treatment'' OR ''Malaria insecticide resistance'' With all of the words Madagascar 2. ''Malaria intervention'' OR ''long lasting insecticidal'' OR ''indoor residual spraying'' OR ''health care'' OR ''health care challenges'' With all of the words Madagascar |
| **Web of Science** | 1. Malaria (Topic) and Malaria transmission (Abstract) or Malaria control (Abstract) or Malaria vector control (Abstract) or Malaria prevention (Abstract) or Malaria diagnosis (Abstract) or Malaria treatment (Abstract) or Malaria insecticide resistance (Abstract) and Madagascar (Title) 2. Malaria (Topic) and Malaria intervention (Abstract) or long lasting insecticidal (Abstract) or indoor residual spraying (Abstract) or health care (Abstract) or health care challenges (Abstract) and Madagascar (Title) |
| **EBSCOhost** | 1. AB (Malaria) AND AB (Malaria transmission) OR AB (Malaria control) OR AB (Malaria vector control) OR AB (treatment) OR AB (vector control) OR AB (insecticide resistance) OR AB (socioeconomic factors) OR AB (healthcare or health care or hospital or health services or health facilities) AND Madagascar 2. AB (Malaria intervention) AND AB (prevention) OR AB (long lasting insecticidal) OR AB (indoor residual spraying) OR AB (health care) OR AB (health care challenges) OR AB (socio-economic factors) OR AB (healthcare or health care or hospital or health services or health facilities) AND Madagascar |
