# Supplementary material for: Understanding inequities in the malaria landscape of Madagascar: a scoping review of current evidence
Source: Malar J. 2026 Jan 14;25:91. doi: 10.1186/s12936-025-05718-7 (PMC12888438; doi:10.1186/s12936-025-05718-7)
Supplement: Supplementary file 2 — Supplementary material 2 Table S2. Quality Assessment Summary [file 12936_2025_5718_MOESM2_ESM.docx]

**Table S2.** Quality Assessment Summary

| Study design | High quality | Moderate quality | Low quality | Total |
| --- | --- | --- | --- | --- |
| Quantitative descriptive | 12 | 1 |  | 13 |
| Quantitative non-randomized | 9 | 3 | 1 | 13 |
| Quantitative randomized controlled trials | 3 | 2 |  | 5 |
| Qualitative | 3 |  |  | 3 |
| Mixed methods | 2 | 3 |  | 5 |
| Not applicable |  |  |  | 5 |
| Total (assessed for quality) | 29(74.4%) | 9(23.1%) | 1(2.5%) | 39 |
| Grand total |  |  |  | 44 |

*Note: Studies marked as ‘‘Not applicable’’ represent guidelines, reports, review, or other document types that do not undergo standard methodological quality evaluation using MMAT criteria*.
