# Supplementary material for: Understanding inequities in the malaria landscape of Madagascar: a scoping review of current evidence
Source: Malar J. 2026 Jan 14;25:91. doi: 10.1186/s12936-025-05718-7 (PMC12888438; doi:10.1186/s12936-025-05718-7)
Supplement: Supplementary file 3 — Supplementary material 3 Table S3. Characteristics and key insights of included studies and reports on malaria control in Madagascar in this review [file 12936_2025_5718_MOESM3_ESM.docx]

**Table S3.** Characteristics and key insights of included studies and reports on malaria control in Madagascar in this review

| No | Author/Year | Study design | Polulation/Sample | Intervention focus | Key outcomes |
| --- | --- | --- | --- | --- | --- |
| *Peer review literatures* | | | | | |
| 1 | Nepomichene et al., 2015 [1] | Entomological survey | Vector populations | Outdoor biting *An. coustani* detection | Identification of new vector; implications for control coverage. |
| 2 | Clouston et al., 2015 [2] | Cross-sectional population survey | Women (15–49 yrs) and children <5 yrs | Socioeconomic determinants | Education and wealth strongly influence malaria knowledge, prevention, and prevalence. |
| 3 | Howes et al., 2016 [3] | Cross-sectional population survey | Routine health facility data (2010–2015) and PfPR maps | Subnational surveillance | Eight ecozones identified; major data gaps (60–75% reporting) affect control planning. |
| 4 | Kesteman et al., 2016 [4] | Nationwide control study | Malaria patients and community controls (2012–2013) | LLIN, IRS, IPTp effectiveness | LLIN and IRS each showed 51% protection; combined use 72%; IPTp 73% effective. |
| 5 | Kesteman et al., 2016 [5] | Nationwide cross-sectional | 15,746 individuals across 62 sites | LLIN, IRS, IPTp, IEC | LLIN PE 41%; IRS community PE 78%; combining LLIN + IRS increased protection; IPTp and IEC effects non-significant. |
| 6 | Kesteman et al., 2016 [6] | Cross-sectional outbreak investigation | 1,615 individuals (20 clusters, SE) | LLIN use, drug supply, environmental factors | Outbreak linked to reduced LLIN integrity and insecticide activity, drug stockouts, and increased rainfall; infection higher among rural, poor, and children aged 6–14. |
| 7 | Mattern et al., 2016 [7] | Qualitative comparative study | 71 semi-structured interviews across 4 sites | Local perceptions of malaria, fever management, bed net use | Revealed socio-cultural inequities: malaria seen as simple fever, not mosquito-borne; biomedical messages poorly aligned with local beliefs; bed net use intermittent and not tied to malaria prevention |
| 8 | Randriamaherijaona et al., 2017 [8] | Experimental hut trial | Local *Anophele*s species, 2 sites | Indoor residual spraying with bendiocarb | Bendiocarb achieved ~80% mosquito mortality up to 5 months; no difference in exophilic/blood-feeding; resistance risk due to long-term use. |
| 9 | Rakotoson et al., 2017 [9] | Bioassays and molecular analysis | Mosquitoes from the field | Insecticides used in ITNs and IRS | Anopheles gambiae showed pyrethroid resistance, but all species were susceptible to pirimiphos-methyl. |
| 10 | Zegers de Beyl et al., 2017 [10] | Evaluation of community-based ITN distribution | 1125 households in Toamasina II District, Madagascar | Community-based ITN distribution pilot | in 96.5% household ownership of ITNs, 81.5% population access, higher ownership among poorer households, and 80% positive feedback on the system. |
| 11 | Raobela et al., 2018 [11] | Therapeutic efficacy study | 348 falciparum malaria patients from 6 sites | Artesunate-amodiaquine (ASAQ) | 99.7% cure rate (day 28) |
| 12 | Howes et al., 2018 [12] | Cross-sectional community survey | Community sample, central Madagascar | Infection prevalence and diagnostic performance | 13.8% PCR vs. 4.1% RDT; most infections were submicroscopic. Village location, bednet ownership, fever, and household infection linked to risk. |
| 13 | Ihantamalala et al., 2018 [13] | Retrospective epidemiological study | 112 health districts in Madagascar (2010–2014) | Malaria incidence data analysis | Increased malaria incidence from 2010 to 2014. High incidence in East/West regions. Seasonal peaks January–July. |
| 14 | Ihantamalala et al., 2018 [14] | Spatial analysis of malaria and mobility data | Central Highlands, Capital, Coastal areas | Malaria transmission mapping | Parasite importation hubs in Central Highlands. Infection sources in coastal areas, seasonal variation |
| 15 | Awantang et al., 2018 [15] | Cross-sectional household survey | Mothers of children under 2 years | Intermittent preventive treatment (IPTp) with sulfadoxine-pyrimethamine (SP) | 11.7% of women received 2+ doses of IPTp. Health provider not offering SP. Community-level factors impacted IPTp coverage. |
| 16 | Girond et al., 2018 [16] | Sentinel surveillance-based study | Malaria cases from sentinel sites (2009–2015) | Mass distribution of LLINs (MDCs) and continuous distribution (CB-CD) | MDCs reduced malaria cases, but effect waned after 1–2 years. |
| 17 | Kang et al., 2018 [17] | Spatio-temporal mapping | Children aged 6–59 months (2011, 2013, 2016) | Malaria Indicator Surveys (MISs) | Malaria prevalence increased since 2011. 42.3% in low-risk areas in 2011, 26.7% in 2016. 9.2% in high-transmission areas by 2016. |
| 18 | Willie et al., 2018 [18] | Performance evaluation of RDT | 260 patient blood samples | SD Bioline Malaria Ag P.f/Pan RDT | Sensitivity: 87%, Specificity: 90% |
| 19 | Mehlotra et al., 2019 [19] | Performance evaluation of RDT | 963 patient samples | SD Bioline Malaria Ag P.f/Pan RDT | Sensitivity varies with parasitemia levels. 16.3% P. falciparum submicroscopic. High parasitemia showed higher RDT band positivity |
| 20 | Randriatsarafara et al., 2019 [20] | Cross-sectional Mixed-Methods Study | Private health facilities across 4 malaria epidemiological zones | RDT-based diagnosis  ACT-based treatment for confirmed cases | Poor adherence to RDT/ACT guidelines;  Low RDT availability/confidence;  Frequent incorrect treatment. |
| 21 | Howes et al., 2019 [21] | Stakeholder workshop and model mapping | 15 stakeholders from institution | Model-based geostatistical malaria maps | Mapped 13 malaria indicators using geostatistics. Workshop used maps to guide malaria control strategies |
| 22 | Nguyen et al., 2020 [22] | Spatiotemporal modelling of malaria seasonality | Data from 2669 health facilities | Health facility data, spatiotemporal model | Mapped malaria seasonality by location. Eastern coast peaks earlier; varying start months by region. Model supports intervention planning |
| 23 | Arambepola et al., 2020 [23] | Bayesian modelling | Health facility data, Malaria Indicator Survey data (2013–2016) | None | Coastal areas had highest prevalence. Seasonal variations observed. Prevalence peaked in 2015. |
| 24 | Arisco et al., 2020 [24] | Cross-sectional study | Four regions in rural Madagascar | None | Aquatic agriculture predicts Anopheles larvae presence. Risk factors differ by region. Ecological and socioeconomic factors influence malaria risk. |
| 25 | Anand et al., 2020 [25] | Mixed methods survey, health facility assessments, provider and volunteer interviews | 35 health facilities, 129 patients, 41 health providers, 34 community health volunteers | Malaria elimination readiness assessment | 25% HFs had no RDTs. 43% of fever patients tested. 75% managers reviewed data. 68% HPs received malaria training. 24% CHVs no longer treated fever. Avg. district readiness score: 52/100. |
| 26 | Steinhardt et al., 2021 [26] | School-based serosurveys | 93 communes, 12,770 students blood samples | Comparison of API with serological data | RDT positivity: 0.5%. Seroprevalence: 17.9% to 59.7%. API identified 71% of high-transmission communes. |
| 27 | Hyde et al., 2021 [27] | Geographic analysis of routine health data (2014–2017) | 73,022 confirmed malaria cases in Ifanadiana District | Adjusted malaria incidence based on underreporting and healthcare access. | Estimated 80% underreporting of malaria cases. Adjusted incidence 4x higher during high transmission season. Improved spatial resolution of malaria surveillance. |
| 28 | Fiadanana et al., 2021 [28] | Secondary analysis of 3 qualitative studies | 192 interviews across 10 districts, children 5–15 years | Assessment of social, cultural, and family determinants of LLIN use | LLINs seen positively but underused by children >5 years. Cultural practices and taboos reduce LLIN use. Younger children prioritized when nets are limited. |
| 29 | Sayre et al., 2021 [29] | Cross-sectional survey | 8050 individuals across 334 households in Farafangana district | Baseline assessment for expanding mCCM to all ages | 25.4% malaria RDT positivity in children <15. Higher prevalence in 5-14 years (31.8%). 28.7% sought care for fever. |
| 30 | Rice et al., 2021 [30] | Cross-sectional survey | 7117 individuals in 1476 households from 31 communities | Malaria prevalence survey in 5 ecological regions | Malaria prevalence varied >10-fold between nearby communities. Highest prevalence in west coast (29.4%). Multiple infections in 50% of households in southeast |
| 31 | Rakotoarisoa et al., 2022 [31] | Randomized controlled trial | 558 children aged 1-15 years from 5 regions | Artemether-lumefantrine (AL) vs Artesunate-amodiaquine (ASAQ | AL cleared gametocytes by day 14; ASAQ took longer (until day 21). AL was faster at gametocyte clearance |
| 32 | Ratovoson et al., 2022 [32] | Cluster RCT | 47,562 individuals from 22 fokontany | Proactive CCM and iCCM | Pro-CCM reduced malaria prevalence in children under 15 (OR = 0.59). |
| 33 | Rogier et al., 2022 [33] | Cross-sectional genotyping | Symptomatic malaria cases (2016–2018) | HRP2/3 antigen screening, pfhrp2/3 genotyping | Prevalence of pfhrp2 gene deletions was 0.6% (95% CI: 0.2%-1.6%), and there were no cases of dual pfhrp2/3 gene deletions observed |
| 34 | Andrianaranjaka et al., 2022 [34] | Pilot Study, Nested-PCR | 170 samples (74 from Ankazomborona, 96 from Matanga) | Used malaria RDTs for DNA extraction | Plasmodium positivity: 23.5%. P. falciparum: 92.2%. P. vivax: 5%. Mixed infection: 2.5%. Multiple infection rate: 28.6% |
| 35 | González et al., 2023 [35] | Quasi-experimental, multicentre evaluation | Rural pregnant women | Community delivery of IPTp (C-IPTp) | IPTp3+ coverage for Madagascar from 17.7% to 40.8%, reflecting a 145.6% increase. |
| 36 | Hilton et al. 2023 [36] | Retrospective observational | 9 districts in Madagascar (2017–2020) | Non-pyrethroid IRS | 30.3% reduction in malaria incidence. Third year IRS reduced cases 30.9% more than first year. 86-90% coverage reduced incidence by 19.7% |
| 37 | Garchitorena et al., 2024 [37] | Cluster randomized tria | 30 health centers, ~1600 households in Farafangana District | mCCM for all ages (intervention) vs mCCM for CU5 only (control) | Care-seeking for fever/malaria nearly tripled. RDTs increased 1.65x in 6-13-year-olds. Larger improvements in remote areas. Significant improvements in health access |
| 38 | Gebreegziabher et al., 2024 [38] | Mixed-methods study | High-risk populations in Antsirabe II, Faratsiho, Antsiranana I districts | Assessment of malaria risk factors and intervention preferences | High-risk groups: rice workers, miners, mobile vendors, students. Risk factors: travel, overnight stays, lack of prevention tools. Barriers: cost, distance to health facilities |
| 39 | Andrianantoandro et al., 2024 [39] | Cost-effectiveness analysis | Two districts in Madagascar (Ankazobe, Brickaville) | IRS and ITNs for malaria control | NMCP cost: USD 45.4 million/year |
| 40 | Ye et al., 2024 [40] | Data quality assessment | Health centers in 2 regions | Malaria data review | Timeliness: 85% (Atsinanana), 95% (Atsimo-Andrefana). Completeness: 43% (Atsinanana), 68% (Atsimo-Andrefana). Source docs: 59% (Atsimo-Andrefana), 48% (Atsinanana) |
| *Grey literatures* | | | | | |
| 41 | Catholic Relief Services, 2020 [41] | Report on multi-sectoral malaria programming | Rural communities in southeastern | Multi-sectoral program combining malaria testing/treatment, nutrition assessment, vector mapping, surveillance, lab analysis, and local health worker capacity building | Malaria hotspots overlap with areas of high malnutrition. Multi-sectoral approaches targeting both malaria and nutrition can improve intervention effectiveness and provide dual benefits. |
| 42 | Ministry of health [42] | Governmental report | Malaria control and prevention strategies | People in Madagascar | LLIN distribution improved; ACT access expanded |
| 43 | WHO 2024 [43] | Global report | Assess malaria burden and progress | Madagascar | High malaria burden, especially in rural areas. ITNs and IRS widely used, but insecticide resistance emerging. Community health workers expanded malaria services. Drug resistance and access gaps remain key challenges. |
| 44 | PMI [44] |  |  |  |  |

*Note: LLIN: Long-Lasting Insecticidal Net, IRS: Indoor Residual Spraying, IPTp: Intermittent Preventive Treatment in Pregnancy, ITN / ITNs: Insecticide-Treated Net(s), C-IPTp: Community Delivery of Intermittent Preventive Treatment in Pregnancy, MDCs: Mass Distribution Campaigns (LLIN distribution), CB-CD: Community-Based Continuous Distribution (of LLINs), iCCM: Integrated Community Case Management, mCCM: Malaria Community Case Management, Pro-CCM / pro-CCM: Proactive Community Case Management, RDT / RDTs: Rapid Diagnostic Test(s), PCR: Polymerase Chain Reaction, Nested-PCR : Nested Polymerase Chain Reaction, API: Annual Parasite Incidence, HRP2/3: Histidine-Rich Protein 2 / 3 (antigens), pfhrp2 / pfhrp3: Plasmodium falciparum histidine-rich protein 2/3 genes, ASAQ: Artesunate, Amodiaquine, AL: Artemether–Lumefantrine, ACT: Artemisinin-based Combination Therapy, SP: Sulfadoxine–Pyrimethamine, CU5: Children Under 5 Years, HFs: Health Facilities, HPs: Health Providers, CHVs: Community Health Volunteers, PfPR: Plasmodium falciparum Parasite Rate, PE: Protective Effect, OR: Odds Ratio, SE: Southeast , WHO – World Health Organization, PMI: President’s Malaria Initiative, NMCP: National Malaria Control Program, CRS: Catholic Relief Services, MIS / MISs: Malaria Indicator Survey(s)*
