# Supplementary material for: Understanding inequities in the malaria landscape of Madagascar: a scoping review of current evidence
Source: Malar J. 2026 Jan 14;25:91. doi: 10.1186/s12936-025-05718-7 (PMC12888438; doi:10.1186/s12936-025-05718-7)
Supplement: Supplementary file 4 — Supplementary material 4 Table S4. Key Findings from the literature on malaria control in Madagascar. This table summarizes the main findings from the studies included in this review, addressing the diverse factors influencing malaria control in Madagascar. It includes data on intervention effectiveness, geographic and seasonal transmission patterns, and socio-economic and health system challenges that impact malaria prevention and treatment efforts across the country [file 12936_2025_5718_MOESM4_ESM.docx]

**Table S4.** Key Findings from the literature on malaria control in Madagascar. This table summarizes the main findings from the studies included in this review, addressing the diverse factors influencing malaria control in Madagascar. It includes data on intervention effectiveness, geographic and seasonal transmission patterns, and socio-economic and health system challenges that impact malaria prevention and treatment efforts across the country

| **No** | **Author/Source**  **(year)** | **Type of document/ literature** | **Summary of key findings** | **Gaps/Challenges identified** | **Relevance to inequities** |
| --- | --- | --- | --- | --- | --- |
| 1 | Nepomichene et al., 2015 [1] | Entomological field survey (cross-sectional, 2013–2014) | *An. coustani* newly implicated as a major vector; showed high abundance (45.2%), Plasmodium infection, and opportunistic indoor/outdoor biting (Highest HBR: 86.1). | Current IRS and ITNs miss outdoor and animal-biting mosquitoes. | *Type of inequity: Geographic and occupational*  Description: Geographic and occupational inequities are evident, as rural highland districts like Ankazobe remain under-served by surveillance and vector control. Indoor-focused interventions miss outdoor-biting *An. coustan*i, and limited surveillance delayed its detection. Poor agro-pastoral households face higher exposure from untreated livestock stables. These findings highlight the need for equity-focused and locally adapted vector control strategies. |
| 2 | Clouston et al., 2015 [2] | Cross-sectional analysis of national survey data (observational study) | Mother’s education and household wealth were strong predictors of malaria knowledge and prevention. Children in the richest households had 75% lower odds of malaria (OR=0.25) vs poorest. Children of mothers with secondary education had 49% lower odds (OR=0.51) vs no education. Significant knowledge gaps existed (e.g., 29% didn’t know fever is a symptom). | Universal bed net distribution reduced wealth-based use gaps, but malaria knowledge and treatment access remain limited, particularly among poorer and less-educated groups. Broad interventions are needed to address these social determinants of vulnerability. | *Type of inequity: Socioeconomic and informational*  Description: A clear socioeconomic gradient exists, with higher wealth and education associated with lower malaria prevalence, indicating that malaria risk is tied to social standing. Poorer households demonstrate lower levels of malaria knowledge and reduced use of preventive measures, heightening vulnerability. Regional disparities in data quality, particularly between districts such as Atsinanana and Atsimo-Andrefana, may further contribute to inequitable resource allocation and intervention targeting. |
| 3 | Howes et al., 2016 [3] | Descriptive epidemiological and operational analysis using routine HMIS data (2010–2015) and geospatial stratification (eco-zones) | Eight ecozones were identified based on *P. falciparum* prevalence and environment. Malaria cases nearly quadrupled from 2010 (~201,000) to 2015 (~739,000), with positivity rising from 33% to 50%. The southeast accounted for 42% of cases but only 16% of the population. Outbreaks (292 between 2012–2015) were concentrated in southern and coastal zones. Reporting improved (55% to 76%), but only ~50% of RDT results were recorded nationally. | Reporting is incomplete (25–50% of facilities), with diagnostic stock-outs, limited outbreak records, weak surveillance, poor health access (56% >5 km from a facility), and no species-specific data. Political instability after 2009 further disrupted control programs and funding. | *Type of inequity: Geographic and health system*  Description: Geographic inequities in malaria burden are evident, with coastal and southern districts experiencing higher prevalence alongside weaker health system coverage. Data gaps and limited healthcare access constrain equitable malaria control efforts, underscoring the need for locally adaptive strategies and strengthened routine surveillance. |
| 4 | Kesteman et al., 2016 [4] | Nationwide Case-Control Study | LLINs: 51% protective effectiveness (PE) nationwide; ineffective in the South (PE −11%).  IRS: 51% PE.  LLIN + IRS: 72% PE.  IPTp: 73% PE, not statistically significant.  Impact: >100,000 clinical cases prevented annually. | LLINs were ineffective in the South, showing local failure.  Short questionnaires, potential recall/selection bias, and low power for IPTp/combined interventions.  IRS effectiveness declined over time, with delays reducing impact. | *Type of inequity: Geographic*  Description: The study highlights a pronounced geographic inequity, as LLINs fail to adequately protect populations in southern Madagascar. This protection gap underscores the need for region-specific malaria control strategies rather than uniform national approaches. |
| 5 | Kesteman et al., 2016 [5] | Nationwide Cross-Sectional Survey | LLINs: 41% protective effectiveness (PE) nationally; completely ineffective (harmful) in the South (OR 4.45).  IRS: Effective at community level (78% PE with >75% coverage), but limited household-level impact.  LLINs + high IRS coverage gave the highest protection (86% PE).  IPTp and IEC: Non-significant protective effects. | Geographic inequity: LLINs failed in the South, representing a major unexplained challenge.  Methodological: Sites centered on health centers; RDTs may miss low-density infections.  Operational: Possible rapid insecticide loss in LLINs, raising questions about distribution frequency. | *Type of inequity: Geographic*  Description: The study reveals a pronounced geographic inequity in intervention effectiveness, as LLINs in southern Madagascar provide limited protection and may even increase malaria risk. This critical gap highlights that national policies fail to protect all regions equally and underscores the need for subnational, context-specific strategies rather than uniform national approaches. |
| 6 | Kesteman et al., 2016 [6] | Outbreak Investigation / Epidemiological and Entomological Study. | The outbreak resulted from multiple failures: increased rainfall, declining bed net use, reduced LLIN insecticidal activity, and stock-outs of antimalarials and RDTs. Parasite prevalence was highest among the poorest tercile (OR 1.54), children 6–14, rural residents (OR 6.25), and those not sleeping under nets regularly (OR 0.51). Waning population immunity after a decade of successful control likely increased outbreak susceptibility. | Fragility of control gains: Sustained efforts are needed, as gains can be quickly lost due to climatic, intervention, and health system failures.  LLIN durability: Nets degraded physically and chemically before their 3-year lifespan, reducing protection.  Health system preparedness: Stock-outs of commodities weakened case management, likely worsening the outbreak. | *Type of inequity: Socioeconomic, geographic, and health system*  Description: The study demonstrates multiple inequities in malaria risk and care. The poorest households faced a disproportionately higher infection burden, while rural residents had six times higher odds of infection compared with urban populations, reflecting disparities in healthcare access and prevention coverage. Frequent ACT and RDT stock-outs further disadvantaged communities reliant on public health facilities, exacerbating inequitable treatment access. |
| 7 | Mattern et al., 2016 [7] | Observational study | Local terminology: ‘‘Tazomoka’’ was rarely used; people said ‘‘Tazo’’ (fever) or ‘‘Tazomahery’’ (strong fever), not linked to mosquitoes.  Care-seeking behavior: Self-medication was first-line; formal care was sought only after 2–3 days if illness worsened.  Bed net use: Nets were mainly for insect nuisance, cold, or privacy, not malaria prevention.  Health messaging: Official malaria messages were largely ineffective; information spread better via informal channels and rumors. | Ineffective communication: National malaria messages clash with local disease concepts and are often ignored or misunderstood.  Cultural and behavioral barriers: Beliefs about disease causation and low perceived severity of “Tazo” limit uptake of preventive measures like LLINs.  Weak health system: Consultations rarely provide effective education, undermining trust and care-seeking. | *Type of inequity: Informational, cultural, behavioral, and health system*  Description: The study highlights deep-rooted inequities arising from ineffective health communication and cultural misalignment. National malaria messages often conflict with local disease concepts, leading to misunderstanding or disregard. Cultural beliefs about disease causation and the perceived mildness of ‘‘Tazo’’ reduce the uptake of preventive measures such as LLIN use. Weaknesses in the health system, where consultations rarely provide effective education, further erode trust and discourage care seeking, perpetuating inequitable malaria outcomes. |
| 8 | Randriamaherijaona et al., 2017 [8] | Quantitatively measures the efficacy of an intervention | Bendiocarb efficacy: Significantly increased mosquito mortality on all substrates (mud, cement, wood, tin, vegetative).  Limitations: Did not reduce blood-feeding or increase mosquito exophily.  Duration: High efficacy (up to 80% mortality) lasted ~5 months.  Vector behavior: Main vectors were zoophilic and exophilic (*An. coustani, An. squamosus)*, limiting IRS impact. | Short residual efficacy: Bendiocarb protection declines after 5 months, requiring multiple costly spray rounds.  Resistance threat: Continued use over 7 years highlights the need for alternative insecticides.  IRS limitation: Main vectors are zoophilic and exophilic, meaning they are less likely to rest indoors, reducing the overall impact and cost-effectiveness of IRS as a standalone intervention. | *Type of inequity: Intervention access and socioeconomic*  Description: The study reveals inequities in intervention effectiveness, as IRS efficacy varies by wall material, such as mud compared with tin, leading to unequal protection across households. These structural differences are closely linked to socioeconomic status, with poorer households often receiving less effective protection. |
| 9 | Rakotoson et al., 2017 [9] | Nationwide Entomological Monitoring | Widespread pyrethroid & DDT resistance: Permethrin resistance confirmed at 4/18 sites; LLIN efficacy threatened in multiple regions.  Key susceptibilities: Vectors remain fully susceptible to bendiocarb and pirimiphos-methyl, making IRS an effective option.  Metabolic resistance: Pre-exposure to PBO/DEF restored susceptibility, indicating metabolic (not genetic) resistance and supporting the use of PBO-treated nets. | Widespread pyrethroid resistance threatens LLIN efficacy, necessitating insecticide rotation and ongoing surveillance, while IPTp coverage remains critically low (11.7%) due to stock-outs and provider practices, highlighting the need for integrated solutions addressing commodities, provider behavior, and community-level norms | *Type of inequity: Geographic*  Description: The study highlights geographic inequity in vector resistance, as populations in areas such as Kiangara and Soavina receive less protection from standard LLINs due to uneven insecticide resistance patterns. |
| 10 | Zegers de Beyl et al., 2017 [10] | Program evaluation / Cross-sectional survey (Observational Study) | The 9-month community distribution (CD) pilot distributed 43,500 nets, raising household ITN ownership to 96.5% (vs. 74.6% without CD). The system effectively reached poorer households and was well accepted, with 84% reporting it was very simple to use. | The long-term sustainability and cost-effectiveness of community-based distribution are uncertain. It depends on strong community networks, training, and logistics, and further research is needed to assess multi-year coverage and household net replacement behavior. | *Type of inequity: health access*  Description: Community-based delivery reached poorer households more effectively, whereas mass campaigns and private-sector channels favored wealthier groups, revealing inequitable access across socioeconomic levels. |
| 11 | Raobela et al., 2018 [11] | Therapeutic efficacy study (clinical trial) | ASAQ remains highly effective: Among 348 patients (6 months–56 years), PCR-corrected day-28 cure rate was 99.7%, with rapid parasite clearance, mild adverse events, significant hemoglobin improvement, and consistent efficacy across sites and years. | Data gaps: No recent comparative data for alternative ACTs (e.g., artemether–lumefantrine).  Resistance detection: Study not powered to detect rare markers (k13 mutations).  Follow-up limitation: Monitoring beyond 28 days was limited.  Access inequities: Drug stock-outs and treatment access disparities were not addressed.  Health system focus: Study assessed efficacy, not delivery or implementation challenges. | *Type of inequity: Health system*  Description: High drug efficacy, indicating that rising malaria incidence is not due to treatment failure, but likely due to inequities in access, supply-chain issues, and surveillance gaps. |
| 12 | Howes et al., 2018 [12] | Cross-sectional community survey (n = 2,143 participants) using microscopy, RDT, and molecular diagnostics. | Overall prevalence: 13.8% (PCR), 4.1% (RDT), 2.4% (microscopy); 82.8% of infections were submicroscopic.  Species prevalence: *P. vivax* (6.6%) and *P. falciparum* (7.0%) similar; 98.5% of P*. vivax* infections were submicroscopic.  Duffy status: Duffy-negative individuals had half the odds of P. vivax infection compared to Duffy-positive (OR 0.52, p < 0.001).  Spatial heterogeneity: Infection varied by health center and village.  Protective factors: Bed net ownership reduced infection risk by ≥50%.  Household risk: Living with an infected member doubled odds of infection for both species. | Relocation of study population (migrants from non-endemic areas) may not represent national demographics. | *Type of inequity: Geographical, Diagnostic, Socioeconomic, Ecological, Surveillance*  Description: Detection capacity and infection prevalence vary across villages and health catchments; RDTs and microscopy under-detect submicroscopic infections, leading to underestimation of malaria prevalence and delayed treatment; migrant rural populations face limited diagnostic access and weaker health infrastructure; rural highland fringe areas exhibit unique transmission patterns, including mixed Plasmodium species and Duffy polymorphisms, not captured nationally; and routine surveillance misses submicroscopic and asymptomatic carriers, creating a hidden parasite reservoir that undermines elimination efforts. |
| 13 | Ihantamalala et al., 2018 [13] | Spatio-temporal analysis of surveillance data | National trend: Malaria incidence increased from 2010 to 2014.  Geographic shift: High-risk clusters moved southward in East and West strata and expanded into Highlands and Fringe zones.  Heterogeneity: Certain districts consistently acted as high-incidence hotspots within broader strata.  Temporal pattern: High-incidence periods (Jan–Jul) were consistent, but outbreak locations shifted over time. | District-level analysis using HMIS data may miss sub-district heterogeneity, is subject to reporting biases, and cannot definitively attribute shifts in malaria clusters to specific causes. | *Type of inequity: Geographical, Programmatic*  Description: The study highlights geographic and programmatic inequities: national-level strata mask local variations, causing uniform strategies to under-serve high-risk hotspots in low-transmission zones and over-serve lower-risk areas in high-transmission zones. Identifying sub-strata clusters is essential for achieving geographic equity in intervention targeting. |
| 14 | Ihantamalala et al., 2018 [14] | Spatial and Mobility Analysis | The central highlands (e.g., Analamanga) are a major sink for imported parasites.  Sources of infection are widespread along the coastal areas, not clustered.  In the capital Antananarivo (a major sink), the primary sources are populated coastal areas.  Parasite importation routes differ from general travel routes. | Coverage limitation: Only 23% of communes have mobile phone data, with gaps in high-transmission areas.  Representativeness: Data from a single provider may not reflect the whole population.  Tracking limitation: Cannot capture individual trips or exact durations.  Data bias: Clinical case data may be underreported and spatially biased.  Seasonality: Transmission seasonality not accounted for. | Type of inequity: Epidemiological, Intervention Targeting  Description: Coastal source populations face endemic transmission and high prevalence, while highland sink areas experience outbreak-driven cases, disproportionately burdening coastal communities. Focusing interventions on highland sink areas neglects source populations, leaving key reservoirs of infection in coastal regions underserved. |
| 15 | Awantang et al., 2018 [15] | Cross-sectional Household Survey | Low IPTp coverage: Only 11.7% of pregnant women received the recommended two doses, revealing a major gap.  Health system barrier: 66.2% of women attending ANC were not offered IPTp, indicating a supply-side failure.  Socioeconomic & gender inequities: Women with primary education were 2.13× more likely to receive IPTp, and participation in health decisions influenced access. | Critical IPTp gap: National coverage is only 11.7%, leaving pregnant women largely unprotected.  Supply-side barriers: Stock-outs and provider practices are the main obstacles, highlighting supply chain and training weaknesses.  Integrated solutions needed: Interventions must address commodities, provider behavior, and community-level gender and social norms. | *Type of inequity: Health system, Socioeconomic, Gender, Geographic*  Description: Pregnant women face multiple inequities in IPTp coverage: drug supply failures and provider practices limit access, less-educated women and those with limited decision-making power are less likely to receive protection even when attending ANC, and access varies significantly by district, showing that location strongly influences coverage. |
| 16 | Girond et al., 2018 [16] | Sentinel Surveillance-Based Epidemiological Study | Mass LLIN campaigns: Highly effective initially (98.2% of sites malaria-alert free in year 1) but protection declines over time (56.7% in year 2; 31.5% in year 3).  Community-based continuous distribution: In one district, reduced malaria cases by 14%, whereas comparable sites without it saw a 12% increase. | Triennial mass LLIN campaigns provide short-lived protection, creating coverage gaps and malaria resurgence, showing that periodic campaigns alone are insufficient for sustained control. | *Type of inequity: Geographic, Socioeconomic, Health access*  Description: Exclusion of low-transmission highlands and limiting pilot continuous LLIN distribution to Toamasina highlights unequal access and higher burden in coastal areas. Waning protection disproportionately affects the poorest households, who cannot afford net replacement and are most vulnerable to malaria-related financial shocks. Reliance on health center data misses marginalized populations with limited access to formal care, further exacerbating inequities. |
| 17 | Kang et al., 2018 [17] | Secondary data analysis using model-based mapping | Malaria prevalence increased markedly from 2011 to 2016: the population in very low-risk areas (<1%) fell from 42.3% to 26.7%, while those in high-transmission areas (>20%) rose from 2.2% to 9.2%. MIS 2016 likely underestimated transmission intensity due to surveys conducted after the peak season. | Survey limitations, low frequency, off-peak timing, coarse spatial resolution, and reliance on model-based extrapolation biased prevalence estimates and limited fine-scale analysis. | *Type of inequity: Geographic, Health access, Socioeconomic, Health system*  Description: High transmission persists in eastern and coastal districts due to ecological vulnerability and weaker health system reach. Remote rural areas are underrepresented in surveys, underestimating malaria burden and reducing intervention prioritization. Coastal high-burden zones overlap with poverty, low education, and poor infrastructure, limiting prevention and care. Diagnostic and reporting capacity is concentrated in urban areas, reducing visibility of malaria burden in remote regions. |
| 18 | Willie et al., 2018 [18] | Diagnostic performance study | RDT sensitivity 87%, specificity 90%; 8 false negatives but no pfhrp2 deletions detected; continued monitoring of RDT efficacy recommended. | False negatives, limited site coverage, lack of continuous gene surveillance, reliance on single RDT type. | Not identified |
| 19 | Mehlotra et al., 2019 [19] | Diagnostic performance study | RDT sensitivity was 87%, and specificity was 90%. False negatives occurred at low parasite densities, though no pfhrp2 deletions were found. | Reduced RDT sensitivity in low-transmission or rural areas causes underdiagnosis and delayed treatment, leading to inequitable malaria control, particularly in remote communities with limited microscopy access. | Not identified |
| 20 | Randriatsarafara et al., 2019 [20] | Cross-sectional Mixed-Methods Study | Provider adherence to national policy for managing uncomplicated malaria (RDT use and ACT prescription).  16.3% of providers had read the malaria case management manual.  55.6% of providers expressed doubts about RDT reliability.  38.2% prescribed antimalarials despite a negative RDT.  50% did not prescribe ACT despite a positive RDT  Non-participation in district health reviews significantly reduced adherence. | Private-sector malaria care faces weak regulation, limited RDT/ACT availability, and a knowledge-practice gap, undermining adherence to national treatment policies. | *Type of inequity: Health system, financial access, and geographic*  Description: The study highlights poor integration and limited training in the private sector, resulting in a two-tiered quality of care (health system inequity). High ACT costs restrict access for poorer patients, driving reliance on cheaper, non-recommended drugs (financial access inequity). Remote providers in sub-desert and highland regions are less likely to use RDTs, widening diagnostic gaps in hard-to-reach areas (geographic inequity). |
| 21 | Howes et al., 2019 [21] | Descriptive implementation study | The study produced geostatistical maps for 13 malaria indicators and trained 15 national stakeholders in their use. Maps revealed strong spatial heterogeneity: ITN ownership was high in coastal areas but low in highlands, and IPTp2+ coverage was under 20% in 40% of districts. These maps enabled better intervention targeting and highlighted local data gaps for decision-making. | Small sample sizes and high predictive uncertainty in some districts limited spatial prediction reliability. Weak health information systems and underuse of spatial data tools constrained integration of model-based evidence into malaria program planning. | *Type of inequity: Geographical, Socioeconomic*  Description: Subnational disparities exist in malaria prevention and care access. Remote and underserved populations, particularly in rural highlands, experience lower access to prevention and treatment services, reflecting overlapping geographic and socioeconomic inequities. |
| 22 | Nguyen et al., 2020 [22] | Spatiotemporal mapping | Most regions peaked Mar–Apr; east coast peaked earlier (Feb). Transmission began in southeast and spread westward. Rainfall and temperature were main drivers. | Incomplete facility coverage, RDT stock-outs, lack of population data, and high uncertainty in remote areas. | *Type of inequity: Geographic, Surveillance*  Description: Regional disparities in malaria risk and data quality highlight the need for localized, equitable control strategies and improved surveillance in underserved regions. |
| 23 | Arambepola et al., 2020 [23] | Spatiotemporal mapping | Monthly malaria prevalence (2013–2016) was highest in southeast and southwest coasts, lowest in highlands and arid south. Prevalence fell in 2014, peaked in 2015 (linked to cyclones and supply disruptions), and stabilized in 2016, with strong seasonality (Feb–Apr). Model was robust despite data uncertainty. | Routine case data were biased by incomplete reporting, limited treatment-seeking, and non-standardized diagnoses. Temporal variation in care-seeking was not captured, some ecological and socioeconomic covariates lacked granularity, and age group mismatches existed between datasets (all ages vs. 6–59 months). | *Type of inequity: Geographic, Surveillance, Health access*  Description: The study highlights regional inequities in malaria burden and data quality, as coastal and rural areas face higher prevalence due to ecological vulnerability and limited access to care. Integrating multiple data sources is essential to address surveillance gaps and guide equitable intervention targeting. |
| 24 | Arisco et al., 2020 [24] | Cross-sectional survey with entomological and epidemiological data | Malaria risk factors varied by region. Aquatic agriculture (e.g., rice paddies) was the strongest predictor of Anopheles larval habitats. Other key factors included remote communities (SE), Anopheles larvae presence (SE, SW), male gender and older age (SE, WC), low bed net use (WC), and low household head education (WC). Bed net use was protective in the West Coast, but coverage was highly inequitable (12.3% in Highlands vs. 95.1% in Southeast). | Larval sampling was limited to a 25 m radius around households, possibly missing distant breeding sites, and low malaria prevalence in regions like the Highlands reduced statistical power to detect risk factors. | *Type of inequity: Geographic, Socioeconomic, Intervention access, Gender/Age*  Description: The study highlights multiple intersecting inequities: remote southeastern communities face higher malaria risk due to geographic isolation; lower education levels increase risk in western and central regions; wide disparities exist in bed net coverage and effectiveness; and males and older children or adults experience higher infection rates. These findings demonstrate that uniform national control strategies are inherently inequitable and must be tailored to local and demographic contexts. |
| 25 | Anand et al., 2020 [25] | Mixed-methods cross-sectional health facility assessment | Average malaria elimination readiness across districts was 52/100. Key gaps included: 25% of health facilities lacked RDTs, only 43% of febrile patients were tested, 68% of providers had malaria training, and 24% of CHVs no longer treated fever. Stock-outs, insufficient training, and funding disruptions were major barriers | Key gaps for malaria elimination include unreliable supply chains (RDT and medicine stock-outs), poor case management (low fever testing rates), insufficient training and supervision of health workers, and limited capacity for outbreak detection and response. | *Type of inequity: Geographic (resources), Health Access*  Description: Health facilities within the same district show large disparities in commodity availability and service readiness, reflecting unequal resource distribution. Community health volunteers (CHVs), essential for reaching remote populations, are frequently undersupplied, with 56 percent lacking malaria commodities, and under-supported, which limits access to timely diagnosis and treatment. |
| 26 | Steinhardt et al., 2021 [26] | Diagnostic Validity Study with Cross-Sectional Serosurvey | low-transmission Central Highlands and Fringe regions, RDT-based prevalence was very low (0.5%), while serological surveys revealed substantial exposure heterogeneity (17.9–59.7%). Routine health facility data (API) identified the top 30% highest-transmission communes with 71% sensitivity and 88% specificity effective in higher-transmission areas but weaker at low levels. Data quality minimally affected performance. School-based serosurveys proved a cost-effective, sensitive tool for detecting hotspots, showing that integrating routine and serological data can enhance equitable and precise IRS targeting. | Data precision Gap: API lacks the accuracy needed for fine-scale stratification, leading to potential misclassification of transmission areas.  Surveillance fragility: Weak health information systems and inconsistent data quality undermine reliable decision-making.  Operational challenge: Balancing the higher cost of serological surveillance with the limited precision of routine, low-cost data remains a major constraint for equitable malaria control. | *Type of inequity: Health access, Intervention targeting, Surveillance*  Description: Populations in remote or underserved areas are systematically excluded from facility-based data, resulting in their malaria burden being underrepresented and under-prioritized in national planning. Reliance on biased routine data, such as API, leads to inequitable allocation of resources like IRS and LLINs, favoring areas with stronger reporting systems rather than those with higher true transmission. School-based serosurveys improve detection but still exclude marginalized groups, such as very young children and those not attending school, perpetuating information gaps that undermine equitable intervention coverage. |
| 27 | Hyde et al., 2021 [27] | Observational cross-sectional sero-epidemiological validation study | A model adjusting routine surveillance data for healthcare utilization improved spatial resolution from 209 km² (health center catchment) to ~20 km² (Fokontany level). It revealed high-incidence clusters in remote, low-elevation areas previously missed by unadjusted data. Passive surveillance captured only ~21% of malaria cases in which four in five infections went unrecorded. Adjusted facility data thus exposed hidden hotspots, enabling more precise local targeting of interventions. | Reliance on paper-based registers led to slow and incomplete data collection. The absence of active surveillance limited validation of adjusted estimates. Underreporting in remote Fokontany required data pooling, reducing spatial precision. Inconsistent recording of RDT stock-outs likely caused underestimation, while unmeasured contextual factors (e.g., proximity to PIVOT-supported centers) may have influenced results. | *Type of inequity: Geographic, Socioeconomic, Health system, Programmatic, Digital*  Description: Over three-quarters of the population live more than an hour from a health facility, leading to under-detection of malaria in remote areas. User fees, transport costs, and poverty reduce care-seeking, skewing surveillance toward wealthier populations. Rural facilities face frequent RDT stock-outs, limited staff, and weak data quality, while surveillance and resource allocation concentrate in accessible areas, leaving marginalized communities underserved. Dependence on paper-based registers and the absence of digital systems such as DHIS2 delay reporting and perpetuate structural data gaps. |
| 28 | Fiadanana et al., 2021 [28] | Secondary analysis of three qualitative studies | LLINs are perceived positively, but their use is low in children aged 5-15 due to: 1) strong prioritization of under-fives and pregnant women; 2) cultural beliefs (LLINs as a symbol of marriage; association with death shrouds); and 3) taboos preventing opposite-sex siblings from sharing a sleeping space. This age group is also more exposed due to evening social activities and sleeping on floors without nets. | A significant gap exists between LLIN distribution and uses in school-aged children, driven by socio-cultural norms and a distribution system based on household headcount that fails to account for actual sleeping arrangements and cultural taboos. | *Type of inequity: Age-Based, Gender-Based, Intra-Household, Geographic/Cultural*  Description: Children aged 5–15 are deprioritized for LLIN use compared to under-fives and pregnant women, resulting in higher malaria risk in this group. Cultural norms associating bed nets with marriage discourage unmarried adolescent boys from using LLINs, increasing exposure. Within households, children sleeping on floors or sofas are less likely to receive LLINs than those in beds, reflecting unequal resource distribution. In the Highlands and West Coast, LLINs are sometimes linked with death, reducing use and creating region-specific cultural barriers to protection. |
| 29 | Sayre et al., 2021 [29] | Cross-Sectional Survey | Malaria prevalence among children <15 years was 25.4%, significantly higher in those aged 5–14 years (31.8%) than in under-fives (16.9%). Care-seeking for fever was low (28.7%) across all ages. Community Health Volunteers (CHVs) provided more accessible care (average travel time 0.48h vs. 1.60h to facilities). Among febrile patients, 71.0% seen by CHVs were tested for malaria, comparable to 87.0% in facilities. Most RDT-positive children (78.6%) were asymptomatic, indicating a large hidden reservoir. | Low numbers of febrile cases reduced statistical power for care-seeking analyses. Findings may not generalize to other Malagasy regions due to transmission heterogeneity. Some inconsistencies occurred, with individuals reporting malaria testing despite not seeking formal care. | *Type of inequity: Geographic, Age-Based, Health Access*  Description: The study reveals a major care gap for school-aged children (5–14 years), who bear the highest malaria burden yet remain excluded from community-based case management. Shorter travel times to CHVs compared to health facilities highlight persistent geographic barriers that disproportionately affect rural populations. Expanding mCCM to all age groups is identified as a key strategy to ensure equitable access to timely malaria diagnosis and treatment. |
| 30 | Rice et al., 2021 [30] | Cross-sectional survey | Malaria prevalence varied over tenfold between nearby rural communities (<50 km apart) with the highest rates on the west coast (up to 46%) and southeast regions. Significant clustering of infections was observed in some households, suggesting local hotspots of transmission. Infection risk was higher among children aged 5–15 years. | Small site-specific samples may overestimate local clustering of cases.  Underreporting and exclusion of asymptomatic infections reduce surveillance accuracy.  Lack of longitudinal and genetic data constrains confirmation of hotspot persistence. | *Type of inequity: Geographic, Socioeconomic, Health access*  Description: The study reveals pronounced spatial and socioeconomic inequities in malaria risk. Rural and forest-edge communities experience the highest exposure but the poorest access to healthcare, underscoring their status as underserved populations that require prioritized and targeted interventions. |
| 31 | Rakotoarisoa et al., 2022 [31] | Therapeutic efficacy study | 558 children (aged 1–15 years) with uncomplicated malaria were randomly assigned to ASAQ or AL. At baseline, 9.7% had detectable gametocytes. Both regimens cleared gametocytes effectively, but AL showed faster clearance, gametocytes undetectable by day 14 versus day 21 for ASAQ. Overall treatment success rate was 100%. | Findings are restricted to children in urban/peri-urban settings, leaving major knowledge gaps on treatment response and transmission risk in rural, high-burden areas with limited ACT access reflecting both geographic and health system inequities. | *Type of inequity: Treatment-related, Ecological*  Description: The study addresses treatment-related determinants of residual transmission, a key barrier to equitable malaria elimination. By confirming ACT efficacy and examining gametocyte clearance, it provides critical insight into the transmission-blocking potential of first- and second-line therapies across diverse ecological regions. |
| 32 | Ratovoson et al., 2022 [32] | Cluster randomized community intervention trial | Malaria prevalence declined more in the intervention arm (8.0% to 5.4%) than in controls (6.8% to 5.7%), with the strongest effect among children <15 years (OR = 0.59, 95% CI ). The combination of pro-CCM and IRS achieved the greatest reduction in malaria prevalence. | Limited effect in adults due to asymptomatic infections and lower care-seeking. Implementation barriers included logistics, supply-chain issues, and CHW workload. Sustainability requires long-term funding and supervision. | *Type of inequity: Geographical, demographic, and operational*  Description: The study shows that malaria persists in remote rural areas with limited access to prevention and care (geographical inequity); school-aged children are often excluded from routine interventions, leaving them at higher risk (demographic inequity); and insufficient CHW coverage with unreliable supply chains weakens service delivery (operational inequity). Proactive community outreach is shown to reduce these inequities and close access and treatment gaps among |
| 33 | Rogier et al., 2022 [33] | Laboratory analysis of clinical samples from Therapeutic Efficacy Studies | Low prevalence of pfhrp2 (0.6%) and pfhrp3 deletions, with no double deletions detected. However, deletions were geographically clustered (3 of 4 from northern Ankazomborona), indicating potential localized diagnostic risk. The combined phenotypic–genotypic surveillance approach proved effective for monitoring RDT reliability using archived samples | The study likely underestimated the true prevalence of pfhrp2/3 deletions because enrollment required a positive HRP2-RDT, automatically excluding double-deletion infections. Additionally, mixed-strain infections could mask deletions, further limiting detection accuracy. | *Type of inequity: Diagnostic and biological inequities.*  Description: The study highlights that even at low prevalence, pfhrp2/3-deleted parasites create unequal access to accurate diagnosis and treatment. Communities such as Ankazomborona face a higher risk of false negative HRP2 RDT results, leading to missed treatment, greater risk of severe disease or death, and continued local transmission, representing a biological inequity driven by diagnostic limitations. |
| 34 | Andrianaranjaka et al., 2022 [34] | Laboratory-based pilot study-Diagnosis | Used RDTs proved a practical DNA source via simple water elution. Nested-PCR detected malaria in 23.5% of samples, predominantly P. falciparum (92.2%). Both high- and low-transmission sites showed high genetic diversity but low multiplicity of infection (MOI = 1.79). Genotyping of msp1, msp2, and glurp revealed numerous distinct alleles, confirming the method’s utility for molecular surveillance. | The study was a pilot with limited samples, reducing its power and generalizability | *Type of inequity: Resource inequity.*  Description: Advanced molecular surveillance remains concentrated in well-funded central laboratories, while remote and resource-poor regions lack access to essential genomic monitoring tools, limiting equitable participation in national malaria surveillance and response. |
| 35 | González et al., 2023 [35] | A quasi-experimental evaluation in four sub-Saharan African countries | IPTp3+ coverage in Madagascar increased from 17.7% to 40.8% (145.6% increase). ANC attendance remained stable, suggesting community delivery improved reach. | Sustainability and supervision of CHWs; variability in local implementation support | Type of inequity: Geographic and health system inequities.  Description: The study highlights that rural pregnant women face barriers to antenatal care attendance and depend heavily on community health workers to access intermittent preventive treatment in pregnancy (IPTp), reflecting unequal access across locations and weaknesses in health system reach to underserved populations. |
| 36 | Hilton et al. 2023 [36] | Retrospective observational study (2017–2021) using national routine health facility data | IRS reduced malaria incidence by 30.3% overall across nine districts. A third consecutive year of IRS achieved an additional 30.9% reduction compared to the first year. Coverage between 86–90% reduced incidence by 19.7%, but effects above 90% were not statistically significant. Sustained IRS produced clear cumulative benefits, especially in high-burden eastern and southern districts. Estimated 116,000 malaria cases averted between 2016–2021. | Accessibility challenges led to low coverage in remote communes. Lack of randomized control limits causal inference. Potential bias in spray reporting and variable health-seeking behavior across rural areas. | *Type of inequity: Geographic, operational, and surveillance inequities.*  Description: The study highlights that remote and hard-to-reach communes consistently failed to meet WHO’s 85% IRS coverage target, leaving them less protected. Dependence on routine data exposes systemic surveillance gaps between well-resourced and peripheral districts, emphasizing the need for equitable and sustained resource allocation to maintain long-term IRS coverage in high-burden rural areas. |
| 37 | Garchitorena et al., 2024 [37] | Cluster-randomized trial (30 health centers, 2019–2021) | Expanding mCCM to all ages markedly improved malaria testing and treatment for individuals >5 years, particularly those >5 km from health centers (RR = 1.21 per km). CHWs conducted most new RDTs, care-seeking and ACT use tripled, and stronger supply chains enhanced access even without full rollout. | Dependence on external support for supply chains and incentives raises sustainability concerns. COVID-19 shortened the intervention, limiting assessment of long-term effects. Effectiveness depends on continuous supply, supervision, and CHW motivation. Despite reduced distance-related gaps, economic and systemic inequities persist. Low fever prevalence limited data precision, highlighting the need for better surveillance. | *Type of inequity: Geographical, age, operational, and socioeconomic*  Description: The study highlights improved access for remote rural populations (geographical inequity), closure of the service gap for individuals over five years who were previously excluded from iCCM (age inequity), exposure of dependence on CHWs and fragile supply systems (operational inequity), and greater reach to poor agricultural households with limited mobility (socioeconomic inequity). Overall, it supports universal, equity-focused malaria service delivery. |
| 38 | Gebreegziabher et al., 2024 [38] | Mixed-methods study on identifying and characterizing high-risk populations in malaria elimination districts in Madagascar | Rice farmers, miners, outdoor/manual workers, and itinerant vendors had significantly higher odds of malaria (ORs 5–55). Mobile vendors and students in urban areas were also at elevated risk due to travel, outdoor work, and low preventive tool use. Barriers included distance, cost of care, lack of LLINs, and poor access to diagnosis. Gatekeepers and local leaders were key to community engagement. | The study found that rice farmers, miners, and itinerant vendors faced the highest malaria risk in low-transmission districts of Madagascar. Outdoor or nighttime work and travel were strongly associated with infection (OR = 5.28–54.9), while LLIN use was protective (OR = 0.44). Spending nights outside or traveling overnight sharply increased risk (OR = 5.89–18.6). Most participants (78%) lacked prevention tools and faced barriers such as distance, cost, and low risk perception. Qualitative data revealed poor LLIN access, reliance on traditional remedies, and minimal outreach to mobile workers; showing that national strategies fail to equitably cover these high-risk, mobile populations. | *Type of inequity: Geographical and occupational*  Description: The study highlights that vulnerable rural and mobile populations face higher malaria risk yet remain underserved by national interventions. It calls for equity-oriented, subnational tailoring through outreach activities, presumptive treatment, and engagement of community gatekeepers to ensure effective coverage of unreached groups. |
| 39 | Andrianantoandro et al., 2024 [39] | Cost-Effectiveness Analysis | The NMCP’s annual cost was USD 2.0 per capita. ITNs were highly cost-effective (USD 45.3–85.4 per DALY), while IRS was much less so (USD 427.6–546.3 per DALY), costing about four times more. In the low-transmission district of Ankazobe, IRS was still implemented despite ITNs being more efficient, highlighting potential inefficiencies in resource allocation. | The study was limited to two of 114 districts, restricting generalizability. Data gaps on private sector care and self-medication reduced completeness. The case-control design could not assess community-level (herd) effects of IRS. Fragmented data systems across partners hindered full national cost tracking by the NMCP. | *Type of inequity: Socioconomic, intervention strategic, and geographic*  Description: The study demonstrates that intervention choices, such as implementing IRS instead of ITNs, affect the fair and efficient use of limited malaria control funds. Deploying the more expensive, less cost-effective IRS in a low-transmission district like Ankazobe represents an inequitable allocation of resources, diverting support from higher-burden areas where ITNs could yield greater impact. This reflects a misalignment between national strategy and local cost-effectiveness, reinforcing both geographic and economic inefficiencies. |
| 40 | Ye et al., 2024 [40] | Data Quality Assessment (routine health system data) | The assessment revealed critical gaps: data element completeness in monthly reports was low (43-68%); reporting accuracy was poor with overreporting of key indicators (Verification Factor >1.1); and data consistency over time was problematic (36-50% of centers outside normal range). Timeliness and report submission completeness were higher. | Major gaps exist in the health information system: a critical lack of written data collection guidelines (available in only 7-14% of centers), insufficient reserve stocks of reporting forms, and inconsistent data entry leading to inaccurate national-level data (DHIS2). Regular data quality assessments are needed. | *Type of inequity: Data and health system*  Description: The study identifies significant disparities in malaria data quality across health regions, with areas such as Atsinanana performing worse than Atsimo-Andrefana on multiple metrics. These inconsistencies risk misallocating resources and driving misguided public health decisions, underscoring the need for equitable data strengthening across regions. |
| ***Grey literature*** | | | | | |
| 41 | Catholic Relief Services, 2020 [41] | Report on multi-sectoral malaria programming | Malaria hotspots overlap with areas of high malnutrition. Multi-sectoral approaches targeting both malaria and nutrition can improve intervention effectiveness and provide dual benefits. | Persistent malaria in nutritionally vulnerable communities. Limited capacity to rapidly identify local drivers of malaria and malnutrition for targeted interventions. | *Type of inequity: Geographic and nutritional*  Description: Remote rural communities with high food insecurity experience higher malaria risk and limited access to prevention and treatment. |
| 42 | Ministry of health [42] | Governmental report | LLIN distribution improved; ACT access expanded | Regional disparities; logistical constraint | None |
| 43 | WHO 2024 [43] | Global report | High malaria burden, especially in rural areas. ITNs and IRS widely used, but insecticide resistance emerging. Community health workers expanded malaria services. Drug resistance and access gaps remain key challenges. | Geographic: Limited malaria control in rural, remote areas.  Treatment Access: Poor access to diagnostics and treatment, especially in underserved regions.  Prevention: Inconsistent coverage of ITNs and IPTp.  Insecticide Resistance: Rising resistance, undermining vector control.  Health System: Weak data quality and health infrastructure.  Community Engagement: Low participation in prevention efforts. | *Type of inequity*: geographic, socioeconomic, gender-related, health system, healthcare access, biological, conflict-related  Description: Malaria prevalence is higher in rural and remote areas, where access to healthcare and prevention is limited. Poorer households experience limited access to malaria prevention and treatment, exacerbating health disparities. Women, particularly pregnant women, face reduced access to treatment and prevention, putting them at higher risk. Barriers such as cost, distance, and lack of services in remote areas limit access to timely malaria diagnosis and treatment. Regional variations in insecticide resistance result in unequal protection across different areas, weakening malaria control efforts.  Ongoing violence and instability disrupt malaria control efforts, leaving displaced populations vulnerable to malaria due to limited access to healthcare and prevention tools. |
| 44 | PMI [44] | Report | Since partnering with PMI, Madagascar has improved malaria coverage, reduced child deaths, and trained over 204,600 health workers. | None | None |

*HBR: Human Biting Rate, MOI: Multiplicity of Infection, msp1, msp2: Merozoite Surface Protein 1 / 2, glurp: Glutamate-rich protein, RDT: Rapid Diagnostic Test, HRP2 / HRP3: Histidine-Rich Protein 2 / 3, pfhrp2 / pfhrp3: Plasmodium falciparum histidine-rich protein genes 2/ 3, PCR: Polymerase Chain Reaction, Nested-PCR: Nested Polymerase Chain Reaction, API: Annual Parasite Incidence, DEF: S,S,S-tributyl phosphorotrithioate (synergist), PBO: Piperonyl Butoxide (insecticide synergist), LLIN(s): Long-Lasting Insecticidal Net(s), IRS: Indoor Residual Spraying, ITN(s): Insecticide-Treated Net(s), CD: Community Distribution, IEC: Information, Education, and Communication, IPTp: Intermittent Preventive Treatment in Pregnancy, ASAQ: Artesunate–Amodiaquine, AL: Artemether–Lumefantrine, ACT: Artemisinin-based Combination Therapy, CHV(s): Community Health Volunteers, CHW(s): Community Health Workers, ANC: Antenatal Care, mCCM / pro-CCM / iCCM:(Malaria) Community Case Management / Proactive CCM / Integrated CCM, HMIS: Health Management Information System, MIS: Malaria Indicator Survey, OR: Odds Ratio, RR: Relative Risk, WC, SE, SW, EC: West Coast, Southeast, Southwest, East Coast (regional labels), DHIS2: District Health Information System 2, HF(s): Health Facility / Health Facilities, NMCP: National Malaria Control Program, PE: Protective Effectiveness, DALY: Disability-Adjusted Life Year, PIVOT: (Health systems partner organization), PMI: President’s Malaria Initiative, CRS: Catholic Relief Services, WHO: World Health Organization.*

1. Nepomichene TNJJ, Tata E, Boyer S. Malaria case in Madagascar, probable implication of a new vector, Anopheles coustani. Malar J [Internet]. BioMed Central Ltd.; 2015 [cited 2025 Nov 8];14:475. https://doi.org/10.1186/S12936-015-1004-9

2. Clouston SAP, Yukich J, Anglewicz P. Social inequalities in malaria knowledge, prevention and prevalence among children under 5 years old and women aged 15–49 in Madagascar. Malar J [Internet]. BioMed Central; 2015 [cited 2025 Nov 8];14:499. https://doi.org/10.1186/S12936-015-1010-Y

3. Howes RE, Mioramalala SA, Ramiranirina B, Franchard T, Rakotorahalahy AJ, Bisanzio D, et al. Contemporary epidemiological overview of malaria in Madagascar: operational utility of reported routine case data for malaria control planning. Malar J [Internet]. BioMed Central; 2016 [cited 2025 Nov 8];15:502. https://doi.org/10.1186/S12936-016-1556-3

4. Kesteman T, Randrianarivelojosia M, Raharimanga V, Randrianasolo L, Piola P, Rogier C. Effectiveness of malaria control interventions in Madagascar: a nationwide case–control survey. Malar J [Internet]. BioMed Central Ltd.; 2016 [cited 2025 Nov 8];15:83. https://doi.org/10.1186/S12936-016-1132-X

5. Kesteman T, Randrianarivelojosia M, Piola P, Rogier C. Post-deployment effectiveness of malaria control interventions on Plasmodium infections in Madagascar: a comprehensive phase IV assessment. Malar J [Internet]. BioMed Central Ltd.; 2016 [cited 2025 Nov 8];15:322. https://doi.org/10.1186/S12936-016-1376-5

6. Kesteman T, Rafalimanantsoa SA, Razafimandimby H, Rasamimanana HH, Raharimanga V, Ramarosandratana B, et al. Multiple causes of an unexpected malaria outbreak in a high-transmission area in Madagascar. Malar J [Internet]. BioMed Central Ltd.; 2016 [cited 2025 Nov 8];15:57. https://doi.org/10.1186/S12936-016-1113-0

7. Mattern C, Pourette D, Raboanary E, Kesteman T, Piola P, Randrianarivelojosia M, et al. “Tazomoka Is Not a Problem”. Local Perspectives on Malaria, Fever Case Management and Bed Net Use in Madagascar. PLoS One [Internet]. Public Library of Science; 2016 [cited 2025 Nov 8];11:e0151068. https://doi.org/10.1371/JOURNAL.PONE.0151068

8. Randriamaherijaona S, Nepomichene TNJJ, Assoukpa J, Madec Y, Boyer S. Efficacy of Bendiocarb Used for Indoor Residual Spraying for Malaria Control in Madagascar: Results With Local Anopheles Species (Diptera: Culicidae) From Experimental Hut Trials. J Med Entomol [Internet]. Oxford Academic; 2017 [cited 2025 Nov 8];54:1031–6. https://doi.org/10.1093/JME/TJX047

9. Rakotoson JD, Fornadel CM, Belemvire A, Norris LC, George K, Caranci A, et al. Insecticide resistance status of three malaria vectors, Anopheles gambiae (s.l.), An. funestus and An. mascarensis, from the south, central and east coasts of Madagascar. Parasit Vectors [Internet]. BioMed Central Ltd.; 2017 [cited 2025 Nov 8];10:396. https://doi.org/10.1186/S13071-017-2336-9

10. de Beyl CZ, Kilian A, Brown A, Sy-Ar M, Selby RA, Randriamanantenasoa F, et al. Evaluation of community-based continuous distribution of long-lasting insecticide-treated nets in Toamasina II District, Madagascar. Malar J [Internet]. 2017 [cited 2025 Nov 8];16:327. https://doi.org/10.1186/S12936-017-1985-7

11. Raobela O, Andriantsoanirina V, Rajaonera DG, Rakotomanga TA, Rabearimanana S, Ralinoro F, et al. Efficacy of artesunate–amodiaquine in the treatment of falciparum uncomplicated malaria in Madagascar. Malar J [Internet]. BioMed Central Ltd.; 2018 [cited 2025 Nov 8];17:284. https://doi.org/10.1186/S12936-018-2440-0

12. Howes RE, Franchard T, Rakotomanga TA, Ramiranirina B, Zikursh M, Cramer EY, et al. Risk Factors for Malaria Infection in Central Madagascar: Insights from a Cross-Sectional Population Survey. Am J Trop Med Hyg [Internet]. American Society of Tropical Medicine and Hygiene; 2018 [cited 2025 Nov 8];99:995. https://doi.org/10.4269/AJTMH.18-0417

13. Ihantamalala FA, Rakotoarimanana FMJ, Ramiadantsoa T, Rakotondramanga JM, Pennober G, Rakotomanana F, et al. Spatial and temporal dynamics of malaria in Madagascar. Malar J [Internet]. BioMed Central; 2018 [cited 2025 Nov 9];17:58. https://doi.org/10.1186/S12936-018-2206-8

14. Ihantamalala FA, Herbreteau V, Rakotoarimanana FMJ, Rakotondramanga JM, Cauchemez S, Rahoilijaona B, et al. Estimating sources and sinks of malaria parasites in Madagascar. Nat Commun [Internet]. Nat Commun; 2018 [cited 2025 Nov 9];9. https://doi.org/10.1038/S41467-018-06290-2

15. Awantang GN, Babalola SO, Koenker H, Fox KA, Toso M, Lewicky N. Malaria-related ideational factors and other correlates associated with intermittent preventive treatment among pregnant women in Madagascar. Malar J [Internet]. Malar J; 2018 [cited 2025 Nov 9];17. https://doi.org/10.1186/S12936-018-2308-3

16. Girond F, Madec Y, Kesteman T, Randrianarivelojosia M, Randremanana R, Randriamampionona L, et al. Evaluating Effectiveness of Mass and Continuous Long-lasting Insecticidal Net Distributions Over Time in Madagascar: A Sentinel Surveillance Based Epidemiological Study. EClinicalMedicine [Internet]. Lancet Publishing Group; 2018 [cited 2025 Nov 9];1:62–9. https://doi.org/10.1016/j.eclinm.2018.07.003

17. Kang SY, Battle KE, Gibson HS, Ratsimbasoa A, Randrianarivelojosia M, Ramboarina S, et al. Spatio-temporal mapping of Madagascar’s Malaria Indicator Survey results to assess Plasmodium falciparum endemicity trends between 2011 and 2016. BMC Med [Internet]. BMC Med; 2018 [cited 2025 Nov 9];16. https://doi.org/10.1186/S12916-018-1060-4

18. Willie N, Mehlotra RK, Howes RE, Rakotomanga TA, Ramboarina S, Ratsimbasoa AC, et al. Insights into the Performance of SD Bioline Malaria Ag P.f/Pan Rapid Diagnostic Test and Plasmodium falciparum Histidine-Rich Protein 2 Gene Variation in Madagascar. Am J Trop Med Hyg [Internet]. Am J Trop Med Hyg; 2018 [cited 2025 Nov 9];98:1683–91. https://doi.org/10.4269/AJTMH.17-0845

19. Mehlotra RK, Howes RE, Cramer EY, Tedrow RE, Rakotomanga TA, Ramboarina S, et al. Plasmodium falciparum Parasitemia and Band Sensitivity of the SD Bioline Malaria Ag P.f/Pan Rapid Diagnostic Test in Madagascar. Am J Trop Med Hyg [Internet]. Am J Trop Med Hyg; 2019 [cited 2025 Nov 9];100:1196–201. https://doi.org/10.4269/AJTMH.18-1013

20. Randriatsarafara FM, Mandrosovololona V, Andrianirinarison JC, Rakotondrandriana AN, Randrianarivo-Solofoniaina AE, Ratsimbasoa A, et al. Adherence of private sector providers to uncomplicated malaria management policy in Madagascar. Pan Afr Med J [Internet]. African Field Epidemiology Network; 2019 [cited 2025 Nov 9];32:79. https://doi.org/10.11604/PAMJ.2019.32.79.14721

21. Howes RE, Hawa K, Andriamamonjy VF, Franchard T, Miarimbola R, Mioramalala SA, et al. A stakeholder workshop about modelled maps of key malaria indicator survey indicators in Madagascar. Malar J [Internet]. Malar J; 2019 [cited 2025 Nov 9];18. https://doi.org/10.1186/S12936-019-2729-7

22. Nguyen M, Howes RE, Lucas TCD, Battle KE, Cameron E, Gibson HS, et al. Mapping malaria seasonality in Madagascar using health facility data. BMC Med [Internet]. BMC Med; 2020 [cited 2025 Nov 9];18. https://doi.org/10.1186/S12916-019-1486-3

23. Arambepola R, Keddie SH, Collins EL, Twohig KA, Amratia P, Bertozzi-Villa A, et al. Spatiotemporal mapping of malaria prevalence in Madagascar using routine surveillance and health survey data. Sci Rep [Internet]. Sci Rep; 2020 [cited 2025 Nov 9];10. https://doi.org/10.1038/S41598-020-75189-0

24. Arisco NJ, Rice BL, Tantely LM, Girod R, Emile GN, Randriamady HJ, et al. Variation in Anopheles distribution and predictors of malaria infection risk across regions of Madagascar. Malar J [Internet]. Malar J; 2020 [cited 2025 Nov 9];19. https://doi.org/10.1186/S12936-020-03423-1

25. Anand A, Favero R, Dentinger C, Ralaivaomisa A, Ramamonjisoa S, Rabozakandraina O, et al. Malaria case management and elimination readiness in health facilities of five districts of Madagascar in 2018. Malar J [Internet]. Malar J; 2020 [cited 2025 Nov 9];19. https://doi.org/10.1186/S12936-020-03417-Z

26. Steinhardt LC, Ravaoarisoa E, Wiegand R, Harimanana A, Hedje J, Cotte AH, et al. School-Based Serosurveys to Assess the Validity of Using Routine Health Facility Data to Target Malaria Interventions in the Central Highlands of Madagascar. J Infect Dis [Internet]. J Infect Dis; 2021 [cited 2025 Nov 9];223:995–1004. https://doi.org/10.1093/INFDIS/JIAA476

27. Hyde E, Bonds MH, Ihantamalala FA, Miller AC, Cordier LF, Razafinjato B, et al. Estimating the local spatio-temporal distribution of malaria from routine health information systems in areas of low health care access and reporting. Int J Health Geogr [Internet]. Int J Health Geogr; 2021 [cited 2025 Nov 9];20. https://doi.org/10.1186/S12942-021-00262-4

28. Njatosoa AF, Mattern C, Pourette D, Kesteman T, Rakotomanana E, Rahaivondrafahitra B, et al. Family, social and cultural determinants of long-lasting insecticidal net (LLIN) use in Madagascar: secondary analysis of three qualitative studies focused on children aged 5-15 years. Malar J [Internet]. Malar J; 2021 [cited 2025 Nov 9];20. https://doi.org/10.1186/S12936-021-03705-2

29. Sayre D, Steinhardt LC, Irinantenaina J, Dentinger C, Rasoanaivo TF, Kapesa L, et al. Baseline malaria prevalence and care-seeking behaviours in rural Madagascar prior to a trial to expand malaria community case management to all ages. Malar J [Internet]. Malar J; 2021 [cited 2025 Nov 9];20. https://doi.org/10.1186/S12936-021-03956-Z

30. Rice BL, Golden CD, Randriamady HJ, Rakotomalala AANA, Vonona MA, Anjaranirina EJG, et al. Fine-scale variation in malaria prevalence across ecological regions in Madagascar: a cross-sectional study. BMC Public Health [Internet]. BMC Public Health; 2021 [cited 2025 Nov 9];21. https://doi.org/10.1186/S12889-021-11090-3

31. Rakotoarisoa MA, Fenomanana J, Dodoson BT, Andrianaranjaka VHI, Ratsimbasoa A. Comparative effect of artemether-lumefantrine and artesunate-amodiaquine on gametocyte clearance in children with uncomplicated Plasmodium falciparum malaria in Madagascar. Malar J [Internet]. Malar J; 2022 [cited 2025 Nov 9];21. https://doi.org/10.1186/S12936-022-04369-2

32. Ratovoson R, Garchitorena A, Kassie D, Ravelonarivo JA, Andrianaranjaka V, Razanatsiorimalala S, et al. Proactive community case management decreased malaria prevalence in rural Madagascar: results from a cluster randomized trial. BMC Med [Internet]. BMC Med; 2022 [cited 2025 Nov 9];20. https://doi.org/10.1186/S12916-022-02530-X

33. Rogier E, McCaffery JN, Nace D, Svigel SS, Assefa A, Hwang J, et al. Plasmodium falciparum pfhrp2 and pfhrp3 Gene Deletions from Persons with Symptomatic Malaria Infection in Ethiopia, Kenya, Madagascar, and Rwanda. Emerg Infect Dis [Internet]. Emerg Infect Dis; 2022 [cited 2025 Nov 9];28:608–16. https://doi.org/10.3201/EID2803.211499

34. Andrianaranjaka VHI, Ravaoarisoa E, Rakotomanga TA, Ralinoro F, Rakoto DAD, Randrianarivo RH, et al. DNA recovery from used malaria RDT to detect Plasmodium species and to assess Plasmodium falciparum genetic diversity: a pilot study in Madagascar. Malar J [Internet]. Malar J; 2022 [cited 2025 Nov 9];21. https://doi.org/10.1186/S12936-022-04246-Y

35. González R, Manun’Ebo MF, Meremikwu M, Rabeza VR, Sacoor C, Figueroa-Romero A, et al. The impact of community delivery of intermittent preventive treatment of malaria in pregnancy on its coverage in four sub-Saharan African countries (Democratic Republic of the Congo, Madagascar, Mozambique, and Nigeria): a quasi-experimental multicentre …. Lancet Glob Health [Internet]. Lancet Glob Health; 2023 [cited 2025 Nov 9];11:e566–74. https://doi.org/10.1016/S2214-109X(23)00051-7

36. Hilton ER, Rabeherisoa S, Ramandimbiarijaona H, Rajaratnam J, Belemvire A, Kapesa L, et al. Using routine health data to evaluate the impact of indoor residual spraying on malaria transmission in Madagascar. BMJ Glob Health [Internet]. BMJ Glob Health; 2023 [cited 2025 Nov 9];8. https://doi.org/10.1136/BMJGH-2022-010818

37. Garchitorena A, Harimanana A, Irinantenaina J, Razanadranaivo HL, Rasoanaivo TF, Sayre D, et al. Expanding community case management of malaria to all ages can improve universal access to malaria diagnosis and treatment: results from a cluster randomized trial in Madagascar. BMC Med [Internet]. BMC Med; 2024 [cited 2025 Nov 9];22. https://doi.org/10.1186/S12916-024-03441-9

38. Gebreegziabher E, Raoliarison A, Ramananjato A, Fanomezana A, Rafaliarisoa M, Ralisata S, et al. Identifying and characterizing high-risk populations in pilot malaria elimination districts in Madagascar: a mixed-methods study. Malar J [Internet]. Malar J; 2024 [cited 2025 Nov 9];23. https://doi.org/10.1186/S12936-024-04927-W

39. Andrianantoandro VTA, Audibert M, Kesteman T, Ravolanjarasoa L, Randrianarivelojosia M, Rogier C. Cost of the national malaria control program and cost-effectiveness of indoor residual spraying and insecticide-treated bed net interventions in two districts of Madagascar. Cost Eff Resour Alloc [Internet]. Cost Eff Resour Alloc; 2024 [cited 2025 Nov 9];22. https://doi.org/10.1186/S12962-024-00598-1

40. Yé M, N’Gbichi JM, Andrianantoandro T, Rabesahala S, Rabibizaka U, Ramiranirina BE, et al. Identifying Strengths and Gaps in Data Management and Reporting through Malaria Routine Data Quality Assessment: Results from Two Health Regions in Madagascar. Am J Trop Med Hyg [Internet]. Am J Trop Med Hyg; 2024 [cited 2025 Nov 9];112:3–9. https://doi.org/10.4269/AJTMH.23-0224

41. Multisectoral Malaria Programming in Madagascar TARGETING MALARIA-MALNUTRITION CO-INTERVENTIONS IN REMOTE COMMUNITIES BACKGROUND. 2020;

42. Rechercher des résultats pour “Tazomoka” | Ministère de la santé publique [Internet]. [cited 2025 Nov 10]. http://www.sante.gov.mg/ministere-sante-publique/?s=Tazomoka. Accessed 10 Nov 2025

43. World Malaria Report 2023. World Health Organization; 2023.

44. President’s Malaria Initiative: Madagascar Country Fact Sheet (Updated February 2024) - Madagascar | ReliefWeb [Internet]. [cited 2025 Nov 10]. https://reliefweb.int/report/madagascar/presidents-malaria-initiative-madagascar-country-fact-sheet-updated-february-2024. Accessed 10 Nov 2025
