# Supplementary material for: Understanding inequities in the malaria landscape of Madagascar: a scoping review of current evidence
Source: Malar J. 2026 Jan 14;25:91. doi: 10.1186/s12936-025-05718-7 (PMC12888438; doi:10.1186/s12936-025-05718-7)
Supplement: Supplementary file 5 — Supplementary material 5 Table S5. Estimated malaria cases and deaths, along with rates per population for global, African countries, and Madagascar in 2023 [file 12936_2025_5718_MOESM5_ESM.docx]

**Table S5.** Estimated malaria cases and deaths, along with rates per population for global, African countries, and Madagascar in 2023

| Region | Population denominator | Estimated cases | Estimated death | Cases burden per capita |
| --- | --- | --- | --- | --- |
| Global | 46784000702 | 263000000 | 597000 | 0.0056 |
| WHO African Region | 1187602333 | 246000000 | 569000 | 0.1274 |
| Madagascar | 31195932 | 3973000 | 15974 | 0.1274 |

*Source: World malaria report 2024*
