# Supplementary material for: Understanding inequities in the malaria landscape of Madagascar: a scoping review of current evidence
Source: Malar J. 2026 Jan 14;25:91. doi: 10.1186/s12936-025-05718-7 (PMC12888438; doi:10.1186/s12936-025-05718-7)
Supplement: Supplementary file 6 — Supplementary material 6 Table S6. Coverage of treatment-seeking behavior for fever, diagnosis rates, and ACT usage among children under 5 years old based on the most recent household survey in Madagascar [file 12936_2025_5718_MOESM6_ESM.docx]

**Table S6.** Coverage of treatment-seeking behavior for fever, diagnosis rates, and ACT usage among children under 5 years old based on the most recent household survey in Madagascar

| Last survey | DHS 2021 |
| --- | --- |
| Treatment seeking for fever | 45.4 (41.8-49.1) |
| Diagnosis among those with fever and for whom care was sought | 40.0 (35.8-44.4) |
| Use of ACTs among those for whom care was sought | 15.0 (12.0-18.6) |
| Use of ACTs among those for whom care was sought and who received a finger or heel prick | 26.9 (20.9-33.8) |
| Use of ACTs among those for whom care was sought and who were treated with an antimalarial | 55.1(46.9-63.0) |

*Source: World malaria report 2024*
