# Supplementary material for: Understanding inequities in the malaria landscape of Madagascar: a scoping review of current evidence
Source: Malar J. 2026 Jan 14;25:91. doi: 10.1186/s12936-025-05718-7 (PMC12888438; doi:10.1186/s12936-025-05718-7)
Supplement: Supplementary file 7 — Supplementary material 7 Table S7. Malaria control funding in Madagascar, 2021–2023 [file 12936_2025_5718_MOESM7_ESM.docx]

**Table S7.** Malaria control funding in Madagascar, 2021–2023

| Funding | | 2021 | 2022 | 2023 |
| --- | --- | --- | --- | --- |
| Contributions reported by donors | Global Fund. | 16 734 951 | 17 398 886 | 58 927 984 |
|  | PMI/USAID2 | 28 835 459 | 26 947 699 | 26 000 000 |
|  | World Bank United Kingdom | - | - | - |
|  | Others | - | - | 19 674 |
| Contributions reported by countries | Government  (NMP) | 7 761 | 16 169 | 11 965 |
|  | Global Fund | 30 712 141 | - | 30 118 000 |
|  | PMI/USAID | - | - | 26 000 000 |
|  | World Bank | - | - | - |
|  | Other  bilateral | - | - | - |
|  | WHO | 45 200 | 74 600 | 100 000 |
|  | UNICEF | - | 199 800 | 284 422 |
|  | other  contributions | - | - | - |

*Source: World malaria report 2024*
