# Supplementary material for: Understanding inequities in the malaria landscape of Madagascar: a scoping review of current evidence
Source: Malar J. 2026 Jan 14;25:91. doi: 10.1186/s12936-025-05718-7 (PMC12888438; doi:10.1186/s12936-025-05718-7)
Supplement: Supplementary file 8 — Supplementary material 8 Table S8. Distribution of commodities and coverage for malaria treatment in Madagascar, 2021-2023, with a focus on ACTs [file 12936_2025_5718_MOESM8_ESM.docx]

**Table S8.** Distribution of commodities and coverage for malaria treatment in Madagascar, 2021-2023, with a focus on ACTs

| Year | 2021 | 2022 | 2023 |
| --- | --- | --- | --- |
| First-line treatments delivered (including ACT) | 1 918 587 | 1 612 781 | 2 689 480 |
| Malaria cases treated with first-line treatments (including ACT) | 1 947 787 | 1 612 781 | 2 689 480 |
| ACT treatment courses delivered | 1 918 587 | 1 612 781 | 2 689 480 |
| Number of malaria cases treated with ACT | 1 9217 55 | 1 612 781 | 2 689 480 |

*Source: World malaria report 2024*
